# PA-DFNet: Polarity-Aware Attention Network with Feature Dynamic Fusion for Point Cloud Classification and Semantic Segmentation

**DOI:** 10.3390/s26134108

**Published:** 2026-06-28

**Authors:** Zhigang Su, Kai Jin, Jingtang Hao, Bing Han

**Affiliations:** Sino-European Institute of Aviation Engineering, Civil Aviation University of China, Tianjin 300300, China; 2024122060@cauc.edu.cn (K.J.); jthao@cauc.edu.cn (J.H.); b-han@cauc.edu.cn (B.H.)

**Keywords:** deep learning, point cloud segmentation, polarity-aware attention, linear attention mechanism, dynamic feature fusion

## Abstract

Point cloud segmentation constitutes a core task in 3D computer vision. However, prevailing models suffer from inherent limitations, including the absence of polarity correlation (i.e., spatial attribute-containing features derived from the separation and calculation of positive/negative correlations within point cloud query–key pairs), inefficient feature fusion, loss of fine-grained geometric details, and excessive computational complexity in self-attention mechanisms. These deficiencies constrain both the performance and practical deployment of such models. To address these challenges, the Polarity-Aware Attention and Feature Dynamic Fusion Network (PA-DFNet) is proposed in this paper. Built upon the PointNet++ framework, PA-DFNet replaces the original Multilayer Perceptron (MLP) with a Polarity-Aware Network (PAN). The PAN enhances key semantic interactions by explicitly separating positive and negative correlations from point cloud query–key pairs, generates adaptive neighborhood weights via integration with a linear attention mechanism, and introduces a learnable power function to perform nonlinear scaling of attention, thereby improving the model’s structural perception capability. Additionally, a Point Cloud Feature Dynamic Fusion (PFF) module is proposed to enable adaptive fusion of encoder–decoder features, preserving rich geometric details. Experimental results demonstrate that, on the ModelNet40 classification task, the overall accuracy (OA) and mean accuracy (mAcc) of PA-DFNet are improved by 2.4% and 2.2%, respectively, compared with PointNet++. On the S3DIS semantic segmentation task, PA-DFNet achieves an mAcc of 72.8% and a mean Intersection over Union (mIoU) of 66.2%, while exhibiting a shorter training time than Point Transformer. In summary, PA-DFNet achieves an optimal balance between segmentation performance and efficiency by effectively controlling the number of model parameters and computational complexity.

## 1. Introduction

Point cloud semantic segmentation technology, which transforms 3D point clouds from raw geometric representations into structured data endowed with rich semantic information, provides fundamental technical underpinnings for high-level tasks in domains such as autonomous driving [1,2], virtual reality [3], and 3D reconstruction [4]. Traditional point cloud segmentation approaches [5,6,7] rely on manually designed heuristics for geometric feature extraction, which are not only labor-intensive and time-consuming to deploy but also necessitate customized development tailored to specific scenarios, thereby rendering them ill-suited to the complex and dynamic practical requirements of real-world applications. With the rapid advancement of deep learning techniques, deep learning-based point cloud semantic segmentation methods, which leverage their efficient processing capabilities to address the inherent irregularity and unordered nature of point cloud data, have emerged as the mainstream research direction in this field.

Early deep learning approaches addressed the unstructured nature inherent to point clouds via indirect processing strategies: specifically, one category transforms point clouds into regular voxel grids [8,9,10] and then employs convolutional neural networks (CNNs) for feature extraction and representation learning, yet such methods suffer from inherent limitations, including challenges in optimizing voxel resolution, prohibitive memory overhead, and ambiguous feature characterization; the other category projects 3D point clouds onto 2D image planes [11,12,13,14], performs semantic segmentation by leveraging well-established 2D computer vision models, and is followed by inverse projection to map the results back to 3D space, but the 2D projection process inevitably incurs loss of critical depth information, thus failing to preserve the intrinsic 3D geometric properties of point clouds.

In recent years, point-based direct processing paradigms have emerged as a prominent research frontier in point cloud semantic segmentation. These paradigms directly perform end-to-end feature learning on raw point cloud data and excavate latent spatial geometric and semantic information through the design of task-specific network architectures. Nevertheless, existing approaches within this paradigm still suffer from three critical bottlenecks that impede further advancement.

First, existing approaches still lack explicit modeling of polarity-aware correlation information in local point cloud neighborhoods. In this work, polarity is defined as spatial attribute-containing features derived from the separation and calculation of positive and negative correlations within point cloud query–key pairs. Polarity correlations are related to both the geometric attributes and semantic distribution of point cloud data. From a geometric standpoint, local geometric variations, such as changes in normal direction, curvature, and surface structure, may provide spatial cues that are reflected as different correlation patterns in the learned feature space; from a semantic perspective, different semantic categories may exhibit relatively weak or negative correlations, whereas points belonging to the same or spatially related categories may show stronger positive correlations. Nevertheless, within the traditional feature extraction paradigm, MLP-based aggregation, ReLU activation, and pooling operations do not explicitly separate and model positive and negative query–key correlations [15]; this may limit the representation of complementary polarity-aware correlation cues, thereby degrading the feature discriminability of point clouds.

Second, a critical limitation of these approaches lies in their low feature fusion efficiency. Mainstream frameworks, exemplified by PointNet++ [16], adopt an encoder–decoder architecture with fixed skip connections. Given the substantial semantic gap between encoder-derived and decoder-derived features, the direct concatenation of these feature representations induces feature incongruities, which impede effective feature fusion and consequently result in the loss of fine-grained geometric details, thereby compromising the segmentation accuracy at object edges.

Third, these approaches are also plagued by prohibitive computational complexity. Although Transformer-based paradigms [17,18] demonstrate superior capability in modeling long-range dependencies, the inherent quadratic complexity of their self-attention mechanism renders them ill-suited for processing large-scale point clouds. Graph convolution-based methods [19] facilitate information propagation via K-nearest neighbor graph construction and introduce the EdgeConv operator, which constructs local neighborhood graphs to generate edge features that characterize the relationships between each point and its neighbors; however, the proliferation of numerous irrelevant connections within these graphs leads to prohibitive computational complexity.

Early research on point-based convolution primarily focused on the point-wise CNN approach [20], which employs fixed convolution kernels to derive weights, thereby compromising network adaptability. Additionally, its normalization strategy incurs unnecessary computational overhead on the network. Flex-convolution [21] constructs convolution kernels using linear functions and achieves neighborhood feature aggregation through interpolation operations; while this enhances flexibility, the limited expressive capacity of linear mapping makes it difficult to capture intricate geometric relationships. PCNN [22] directly assigns convolution kernel weights to individual points and computes feature interactions via correlation functions; however, it lacks a neighborhood selection mechanism, leading to redundant computations and feature ambiguity. Reference [23] introduces the kernel point convolution (KPConv) framework, which incorporates both rigid and deformable convolution modules. Rigid convolution computes the weight matrix for each kernel point by calculating the distance between the central point and its neighboring points, and subsequently generates per-point features through kernel point convolution, enabling arbitrary configuration of kernel point counts and providing greater flexibility compared to fixed-kernel counterparts (e.g., point-wise convolution). PAConv [24] further improves point convolution by dynamically assembling convolution kernels from a weight bank according to relative point positions, representing a position-adaptive kernel generation strategy. In contrast, the proposed PAN focuses on polarity-aware query–key correlation decomposition in local neighborhoods, where adaptive kernels are guided by positive and negative semantic correlations rather than only by relative geometric positions. Although these methods have effectively enhanced 3D point cloud segmentation performance, they all demonstrate limited capacity to model complex geometric structures, as they overlook negative semantic correlations among points. Furthermore, the weight assignment mechanisms of contemporary point convolution methods prioritize positive semantic correlations (e.g., similarity-based aggregation of neighboring points), while disregarding negative semantic correlations (such as repulsive constraints for dissimilar points), leaving significant room for further refinement.

Notably, diffusion probabilistic network (DPN)-based paradigms, which have emerged in recent years, offer a novel perspective for point cloud processing. These methods learn the underlying data distribution via a progressive pipeline of forward noise injection and reverse denoising, exhibiting superior capabilities in modeling intricate geometric structures and bolstering noise robustness. For instance, Chang Liu et al. [25] leveraged diffusion models to augment point cloud features, thereby boosting the discriminability of fine-grained local geometric details; conversely, Yangdong Chen et al. [26] integrated diffusion models with pre-training enhancement techniques to facilitate sketch-driven point cloud generation. However, DPN-based methods still suffer from notable limitations in point cloud segmentation tasks: their iterative denoising process prioritizes global distribution fitting over targeted modeling of polarity correlations; multi-scale feature fusion relies on static, inflexible strategies, which hinders the adaptive recalibration of weights for features across different hierarchical levels; and the iterative mechanism of reverse denoising introduces prohibitive computational overhead, resulting in diminished efficiency when handling large-scale point clouds, thus posing the same fundamental challenge of balancing computational complexity and model performance.

To mitigate the aforementioned challenges, a novel point cloud processing network (Polarity-Aware Attention and Feature Dynamic Fusion Network, PA-DFNet) is proposed in this paper, constructed upon the PointNet++ framework. Through the design of innovative, task-specific modules, PA-DFNet attains a well-balanced trade-off between performance and efficiency. It should be noted that the main innovation of this work does not lie in the general concept of polarity modeling alone. Instead, the novelty lies in adapting polarity-aware query–key correlation decomposition to unordered local point cloud neighborhoods, converting the learned polarity-aware correlations into adaptive convolution kernels for local feature aggregation, and integrating PAN with PFF into a unified framework for point cloud classification and semantic segmentation. The key contributions of this research are outlined as follows:

1. A Polarity-Aware Network (PAN) is proposed to replace the MLP in PointNet++. PAN adapts polarity-aware query–key correlation decomposition to K-nearest-neighbor point cloud neighborhoods and generates adaptive convolution kernels for local feature aggregation. By combining linear attention with a learnable power function, PAN enhances the modeling of complementary positive and negative correlation cues while maintaining computational efficiency.
2. A Point Cloud Feature Dynamic Fusion (PFF) module is introduced, which adaptively recalibrates the multi-scale feature weights of encoders and decoders via a synergistic combination of channel and spatial attention mechanisms. In contrast to the static, fixed skip connections in PointNet++, the PFF module dynamically modulates the weights of features across different scales, effectively retaining fine-grained geometric details and boosting segmentation accuracy.
3. A well-optimized trade-off between efficiency and performance is achieved. The polarity-aware linear attention mechanism circumvents the quadratic complexity inherent to traditional self-attention, facilitating substantial improvements in point cloud classification and semantic segmentation performance while keeping the number of model parameters and computational overhead in check.

The remainder of this paper is structured as follows: Section 2 presents a comprehensive review of related work; Section 3 details the proposed methodological framework; Section 4 outlines the overall network architecture; Section 5 reports the experimental results and corresponding in-depth analysis; Section 6 concludes the paper and delineates promising future research directions.

## 2. Related Works

As a foundational core task in 3D computer vision, point cloud semantic segmentation has witnessed a proliferation of deep learning-driven methodologies in recent years. we delineate the key distinctions between the proposed methodology and existing state-of-the-art works.

### 2.1. Unsupervised Point Cloud Segmentation Methods

Unsupervised and weakly supervised methods lower the barrier to the practical deployment of point cloud segmentation by reducing reliance on annotated data, and their core principle centers on leveraging the inherent structural properties of data to mine semantic consistency. Qin et al. [27] proposed the Point Domain Adaption Network (PointDAN) model, which dynamically modulates the receptive field via an adaptive node module and achieves cross-object feature alignment by integrating attention mechanisms with adversarial training, thereby establishing an effective structural modeling paradigm for the unsupervised domain. However, this method demonstrates limited capability to capture fine-grained local geometric details, thereby rendering it less effective for the semantic segmentation of complex-shaped objects.

To address the insufficiency of local geometric learning, the Multi-Angle Point Cloud Variational Autoencoder (MAP-VAE) [28] is proposed as a multi-angle variational autoencoder, which explicitly models the spatial correlations between local structures and global shapes through joint learning of global and local geometric features, thereby bolstering semantic consistency in unsupervised scenarios. Nevertheless, its reliance on predefined angle partitions leads to limited adaptability to irregularly shaped objects.

Chen et al. [29] proposed a concise and interpretable unsupervised method that originates from the inherent encoding of 3D structures. This approach facilitates cross-instance transfer of semantic labels by mining structural similarities yet exhibits limited discriminative capability for categories characterized by significant topological discrepancies. Rao et al. [30] introduced a Global–Local Bidirectional Reasoning (GLBR) framework, which infers global structural configurations from local patterns while constraining local geometric shapes via global structural properties. This further reinforces the intrinsic correlation between semantic information and geometric features.

From the above analysis, existing unsupervised paradigms still suffer from notable limitations, including constrained segmentation accuracy and a narrow application scope, thereby rendering them incapable of replacing supervised learning methodologies in complex indoor and outdoor scenarios.

### 2.2. Research on Linear Attention Mechanism

The attention mechanism enhances feature discriminability by explicitly modeling the dependency relationships between data points. Consider a point cloud sequence $X\in R^{N\times C}$ consisting of *N* samples, where *C* denotes the feature dimension of each point. The $O(N^{2})$ complexity of traditional self-attention mechanisms severely restricts their applicability in large-scale point cloud processing. In contrast, linear attention mechanisms approximate Softmax normalization via a kernel function ϕ(·), reducing the computational complexity to O(N), thereby enabling efficient processing of point cloud data.

The fundamental unit in attention computation is typically represented as a token, and in this work, each individual point corresponds to one token. If the input sequence X is partitioned into *h* heads along the feature dimension, with each head having a dimension *d*, then C=h×d. By applying the attention mechanism to each head, long-range dependencies within each head are captured, and the corresponding attention output matrix for the head is given by

(1) $$O=\mathrm{Softmax}\left(\frac{QK^{⊤}}{\sqrt{d}}\right)V$$

where $Q\in R^{N\times d}$, $K\in R^{N\times d}$, and $V\in R^{N\times d}$ denote the query, key, and value matrices, respectively, each of size N×d, with *d* being the feature dimension per head. At this stage, the computational complexity of each attention head amounts to $O(N^{2}d)$, which renders traditional self-attention mechanisms inefficient for long-sequence data processing and limits their capability to handle large-scale point cloud datasets.

Katharopoulos et al. [31] first introduced the linear attention framework, which leverages a kernel function ϕ(·) to approximate the Softmax normalization operation in Equation (1) and restructures the attention computation pipeline via the associative law of matrix multiplication. Let the *d*-dimensional row vectors $q_{r}$, $k_{r}$, $v_{r}$, and $o_{r}$ denote the r-th row vectors of the query (Q), key (K), value (V), and output (O) matrices, respectively. After enhancing the query–key pairs using the kernel function ϕ(·), the attention output for the r-th row can be expressed as

(2) $$o_{r}=\frac{\sum_{j=1}^{N}\phi \left(q_{r}\right)\phi {\left(k_{j}\right)}^{⊤}v_{j}}{\sum_{j=1}^{N}\phi \left(q_{r}\right)\phi {\left(k_{j}\right)}^{⊤}}=\frac{\phi \left(q_{r}\right)\sum_{j=1}^{N}\phi {\left(k_{j}\right)}^{⊤}v_{j}}{\phi \left(q_{r}\right)\sum_{j=1}^{N}\phi {\left(k_{j}\right)}^{⊤}}$$

From Equation (2), it can be observed that, by leveraging the associative law of matrix multiplication, the kernel features of all key–value pairs are pre-aggregated first, and each query $q_{r}$ then interacts only with these pre-aggregated results, reducing the computational complexity of each attention head from $O(N^{2}d)$ to $O(Nd^{2})$. This approach improves computational efficiency through the linear approximation of kernel functions. However, the introduction of such linear approximation also compromises the model’s capability to capture semantic correlations.

Subsequent research efforts have strengthened the capacity to capture semantic correlations by refining kernel function designs. Shen et al. [32] employed ReLU(·) as the kernel function, which reinforces the nonlinear interactions of local features; Katharopoulos et al. [31] utilized the 1+ELU(·) formulation, preserving negative value information via an exponential linear unit (ELU). Choromanski et al. [33] proposed Performer, which approximates Softmax attention using positive orthogonal random features and achieves linear attention computation. Qin et al. [34] introduced the Cosformer, which integrates a cosine function kernel and a reweighting mechanism to enhance local inductive bias, yielding superior performance in long-sequence tasks.

### 2.3. PointNet++ Model

**Figure 1:**
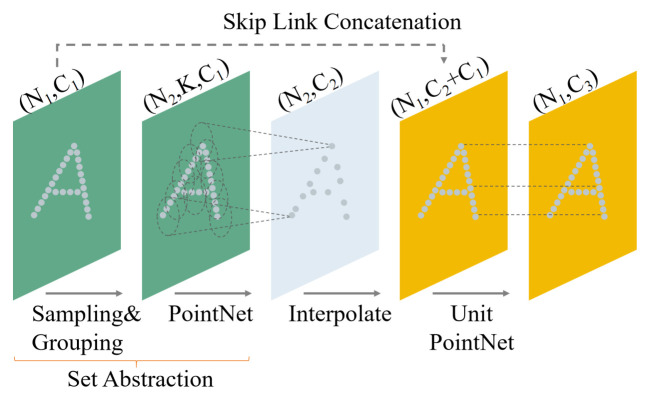
Feature aggregation through set abstraction and skip connections in PointNet++.

The approach proposed in this paper is built upon the PointNet++ [16] framework—a representative backbone in 3D point cloud semantic segmentation, which incorporates targeted refinements to the original PointNet architecture. The core strength of PointNet++ resides in its hierarchical feature learning paradigm, which enables the efficient extraction of both fine-grained local and holistic global point cloud representations. Through a sequence of key operations, including sampling and grouping, feature aggregation, feature interpolation, skip connections, and point-wise nonlinear transformations, it incrementally learns more discriminative global features from low-level local feature encodings. The detailed workflow is illustrated in Figure 1.

Recent studies have increasingly emphasized that effective point cloud segmentation depends not only on local geometric descriptors but also on richer contextual representation learning. Peng et al. [35] introduced external point-set context learning for point cloud segmentation, where auxiliary point-set information is exploited to enhance contextual discrimination beyond the target point set itself. This line of research indicates that the semantic identity of a point is often determined by its interaction with surrounding or external structural cues rather than by isolated local features.

In addition, intelligent point cloud understanding has recently expanded from conventional classification and segmentation toward sensing-oriented 3D perception, compression, completion, reconstruction, quality assessment, and cross-modal comprehension. Wang [36] summarized recent advances in intelligent point cloud processing, sensing, and comprehension, highlighting the importance of robust feature representation, efficient processing, and task-aware 3D perception. These trends are consistent with the design of PA-DFNet: PAN enhances local feature interaction and structural discrimination, whereas PFF adaptively recalibrates multi-scale encoder–decoder features to bridge semantic gaps and retain fine-grained geometric information.

## 3. PAN Model

The PAN module is designed to address the limitations of polarity correlation loss and inadequate structural representation inherent to traditional MLPs in point cloud feature extraction. By decoupling the positive and negative correlations of query–key pairs within point cloud geometric features, this module constructs convolution kernels that explicitly encode polarity-aware correlations, and incorporates a linear attention mechanism to reduce computational complexity, ultimately enabling the modeling of negative query–key correlations and enhancing the discriminative perception of local geometric structures.

### 3.1. PAN Module Overall Architecture

**Figure 2:**
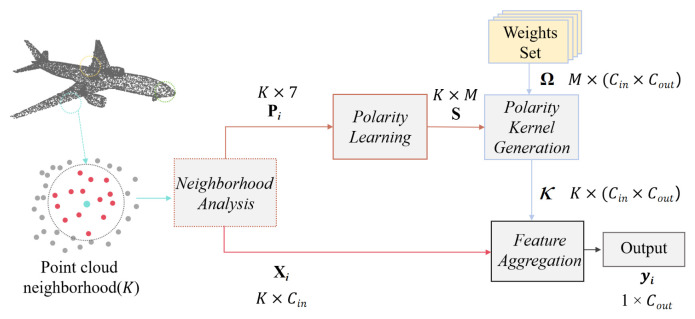
Polarity-Aware Network model.

The core design of the PAN module is illustrated in Figure 2, which depicts the process by which the PAN module operates on the K-nearest neighbors of a point in the point cloud, completing the local feature aggregation processing for that point.

For any point $p_{i}$ in the point cloud, referred to as the central point, the PAN module aggregates local information from its *K* nearest neighbors through four sequential sub-modules: neighborhood analysis, polarity learning, polarity convolution kernel generation, and feature aggregation. The neighborhood analysis sub-module first constructs the geometric feature matrix $P_{i}$ and the semantic feature matrix $X_{i}$ for the local neighborhood. The polarity learning sub-module then estimates a combination coefficient matrix $S\in R^{K\times M}$ by modeling the positive and negative correlations between the central point and its neighboring points. Based on S and the learnable neighborhood weight set ω, the polarity convolution kernel generation sub-module produces adaptive convolution kernels for different neighboring points. Finally, the feature aggregation sub-module applies these kernels to the corresponding semantic features and sums the results to obtain the aggregated feature of the central point.

### 3.2. Neighborhood Analysis

Given the central point $p_{i}$ and its *K* nearest neighbors, the purpose of neighborhood analysis is to construct two local representations: the neighborhood geometric feature matrix $P_{i}$ and the semantic feature matrix $X_{i}$. Instead of repeatedly describing the whole aggregation process, this subsection focuses on the mathematical definition of the geometric descriptor used for each neighboring point.

Let the spatial coordinates of the central point $p_{i}$ and a neighboring point $p_{j}$ be $\left(x_{i},y_{i},z_{i}\right)$ and $\left(x_{j},y_{j},z_{j}\right)$, respectively. The geometric descriptor of $p_{j}$ is defined by concatenating the absolute coordinates of the central point, the relative displacement from $p_{i}$ to $p_{j}$, and their Euclidean distance:

(3) $$p_{ij}=\left[x_{i},y_{i},z_{i},x_{j}-x_{i},y_{j}-y_{i},z_{j}-z_{i},d_{ij}\right]$$

where $d_{ij}={∥p_{j}-p_{i}∥}_{2}$ denotes the Euclidean distance between $p_{j}$ and $p_{i}$. By applying Equation (3) to all *K* neighboring points, the neighborhood geometric feature matrix $P_{i}\in R^{K\times 7}$ is obtained. The semantic feature matrix $X_{i}\in R^{K\times C_{\mathrm{in}}}$ consists of the feature vectors of the same *K* neighboring points, where $C_{\mathrm{in}}$ denotes the input feature dimension of the current layer. Together, $P_{i}$ and $X_{i}$ provide the geometric and semantic inputs for the subsequent polarity learning sub-module.

### 3.3. Polarity Learning

**Figure 3:**
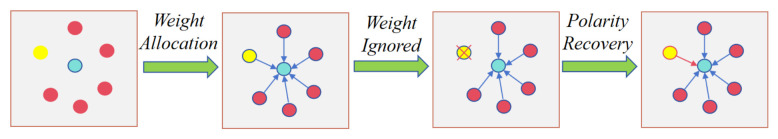
Polarity learning mechanism. The red points represent positive polarity with respect to the central point, while the yellow points represent negative polarity with respect to the central point.

The polarity learning sub-module within the PAN module incorporates polarity correlation information into the attention computation pipeline via a dedicated polarity-aware learning mechanism, enabling the separate modeling of positive and negative semantic correlations. This effectively addresses the limited feature discriminability of conventional linear attention mechanisms, an issue rooted in their neglect of negative semantic correlations. The process by which the polarity learning mechanism extracts positive and negative semantic correlations within the local neighborhood is explicitly divided into four stages, as illustrated in Figure 3.

In the initial stage of the polarity learning mechanism, no feature aggregation has been performed on neighboring points. Consequently, the weights in the neighborhood weight set exhibit a uniform distribution with no distinct polarity differences. At this point, the processing pathways corresponding to positive and negative semantic correlations remain inactive, rendering the model unable to effectively capture the inherent geometric polarity of point clouds (e.g., discrepancies in normal vector directions) and semantic opposition (e.g., the spatial attribute differences between “wall” and “floor” contexts).

In the positive bias stage, constrained by traditional feature aggregation mechanisms, the model prioritizes positive semantic correlations between spatially proximal neighboring points. In contrast, negative semantic correlations are entirely overlooked. As depicted in Figure 3, the weight of the negative pathway approaches 0. This one-sided bias prevents the model from discerning polarity differences between semantic categories and geometric structures, ultimately degrading feature discriminability.

Next, transitioning to the negative weight learning stage, the model performs targeted weight optimization for the processing pathways corresponding to negative semantic correlations. The weight of the negative pathway gradually increases from a near-zero state, initially establishing a polarity distinction relative to the positive pathway, thereby laying a core foundation for subsequent enhancements in the representation of complementary polarity-aware correlations.

Finally, entering the polarity recovery stage, negative and positive weights form a collaborative distribution (as indicated by the differently colored arrows pointing to the central point in Figure 3). Notably, the negative pathway can accurately match and model negative semantic correlations; this not only explicitly characterizes positive and negative correlation cues but also enhances the capability to capture fine geometric structures (e.g., edges and corners) through the synergistic reinforcement of positive and negative pathways, significantly improving the completeness and accuracy of local feature extraction for point clouds.

**Figure 4:**
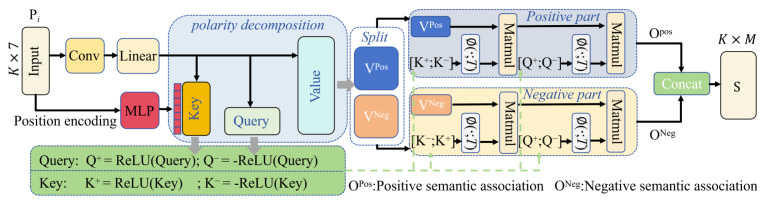
Composition of the polarity learning sub-module.

Polarity decomposition enables efficient and comprehensive feature correlation modeling by decoupling positive and negative correlations and integrating a learnable scaling function [37]. The detailed workflow is illustrated in Figure 4. The polarity learning sub-module comprises four core components: linear attention matrix generation, polarity decomposition, dual-branch semantic correlation learning, and fusion output.

The generation of the linear attention matrix involves converting the input neighborhood geometric features $P_{i}$ of point $p_{i}$ into the query matrix Q, key matrix K, and value matrix V required by the linear attention mechanism. To enable the network to learn hierarchical representations that progress from simple geometric features to complex semantic features, dimension enhancement is first performed via multilayer convolution to extract hierarchical features:

(4) $$H_{l}=\left\{\begin{array}{cc} \mathrm{ReLU}\left({\mathrm{BN}}_{l}\left({\mathrm{Conv}}_{l}\left(P_{i}\right)\right)\right), & l=1\\ \mathrm{ReLU}\left({\mathrm{BN}}_{l}\left({\mathrm{Conv}}_{l}\left(H_{l-1}\right)\right)\right), & l<L\\ {\mathrm{Conv}}_{l}\left(H_{l-1}\right), & l=L \end{array}\right.$$

where $H_{l}$ denotes the output feature of the *l*-th layer, and ${\mathrm{Conv}}_{l}$ and ${\mathrm{BN}}_{l}$ correspond to the 1 × 1 convolution and batch normalization operations of the *l*-th layer, respectively. Except for the final layer, the ReLU activation function is employed to introduce nonlinearity, and *L* is the total number of convolution layers. Experimental results on different datasets demonstrate that the network performance is optimal when L=4.

After the convolution operation, a linear transformation layer is introduced, which consists of shared learnable linear transformation matrices $W_{Q}$, $W_{K}$, and $W_{V}$. Together with the output feature $H_{L}$ from the final convolution layer, these matrices form the query matrix Q, key matrix K, and value matrix V required for linear attention:

(5) $$Q=H_{L}\cdot W_{Q},\;K=H_{L}\cdot W_{K},\;V=H_{L}\cdot W_{V}$$

where the dimensions of Q, K, and V after linear transformation are all K×M. With this, the query matrix Q, key matrix K, and value matrix V required for attention computation are fully constructed. The primary objective of the polarity decomposition part is to decompose the query matrix Q and key matrix K into positive and negative sub-matrices that capture polarity correlations. First, to enhance the richness of semantic representations between neighboring regions, a learnable MLP is introduced to encode positional features of the point cloud:

(6) $$\delta_{j}=\mathrm{MLP}\left(p_{j}-p_{i}\right)$$

where the MLP consists of two linear layers followed by a ReLU activation layer. Subsequently, the positional encoding feature $\delta_{j}$ is combined with the corresponding row vector of the key matrix K to form $k_{\delta j}=k_{j}+\delta_{j}$, which injects structured spatial information to support subsequent attention computation. The key matrix integrated with this positional encoding feature is denoted as $K_{\delta }$. Matrix $K_{\delta }$ is then split into two sub-matrices based on element-wise polarity: the positive component matrix $K^{+}$ (derived via ReLU activation on $K_{\delta }$) and the negative component matrix $K^{-}$ (derived via ReLU activation on −$K_{\delta }$), as formulated below:

(7) $$K^{+}=\mathrm{ReLU}\left(K_{\delta }\right),\;K^{-}=\mathrm{ReLU}\left(-K_{\delta }\right)$$

The relationship between the integrated key matrix $K_{\delta }$ and its decomposed positive ($K^{+}$) and negative ($K^{-}$) components is given by

(8) $$K_{\delta }=K^{+}-K^{-}$$

An analogous decomposition is applied to the query matrix Q, splitting it into a positive component matrix $Q^{+}=\mathrm{ReLU}\left(Q\right)$ and a negative component matrix $Q^{-}=\mathrm{ReLU}\left(-Q\right)$. Correspondingly, the row vectors of Q and $K_{\delta }$ can be re-expressed as

(9) $$q_{j}=q_{j}^{+}-q_{j}^{-},\;k_{\delta j}=k_{\delta j}^{+}-k_{\delta j}^{-}$$

where $q_{j}^{+}$ and $k_{\delta j}^{+}$ denote the positive components of the respective vectors, while $q_{j}^{-}$ and $k_{\delta j}^{-}$ represent their corresponding negative components. The value matrix V serves as the data matrix for semantic correlation computation. To enable independent calculation of positive and negative semantic correlations in subsequent steps, V is split evenly along the feature dimension, yielding $V=[V^{L},V^{R}]$. Here, $V^{L}$ and $V^{R}$ are, respectively, assigned to positive and negative semantic correlation calculations, each with a dimension of K×(M/2). The semantic correlation learning part comprises two parallel structures (for positive and negative semantic correlation learning), as depicted in Figure 4. The core step of semantic correlation learning is to quantify the semantic correlation weight via the inner product of the query vector $q_{j}$ and the key vector $k_{\delta j}$:

(10) $$\langle q_{j},k_{\delta j}\rangle =\langle q_{j}^{+},k_{\delta j}^{+}\rangle +\underset{\mathrm{neglected}\;\mathrm{negatives}}{\underset{︸}{\langle q_{j}^{-},k_{\delta j}^{-}\rangle -\langle q_{j}^{+},k_{\delta j}^{-}\rangle -\langle q_{j}^{-},k_{\delta j}^{+}\rangle }}$$

In Equation (10), the first two terms on the right-hand side capture positive semantic correlations (e.g., the aggregation of structurally similar regions), while the latter two terms characterize negative semantic correlations (e.g., the differentiation of structurally opposing regions). Traditional attention mechanisms only consider $q_{j}^{+}$ and $k_{\delta j}^{+}$, treating $q_{j}^{-}$ and $k_{\delta j}^{-}$ as zero vectors. Consequently, only the first term in Equation (10) is retained, and all other terms are overlooked. In contrast, polarity-aware attention separates query–key pairs by polarity and computes their interactions independently. By restoring the inner products $\langle q_{j}^{-},k_{\delta j}^{-}\rangle$, $\langle q_{j}^{+},k_{\delta j}^{-}\rangle$, and $\langle q_{j}^{-},k_{\delta j}^{+}\rangle$, it strengthens point–pair correlations and resolves the issue of missing negative semantic correlations. Moreover, the presence of $\langle q_{j}^{-},k_{\delta j}^{-}\rangle$ supplements positive semantic correlations. Consequently, the polarity-aware attention mechanism exhibits stronger feature perception capabilities compared to traditional attention mechanisms. The quantified semantic correlation weights require normalization via the Softmax function. To reduce computational complexity while preserving the capability of polarity-aware semantic modeling, this study adopts the linear attention mechanism based on kernel functions ϕ(·) (following the approach in [32]) to approximate attention computations within point cloud neighborhoods. The attention weight calculation is updated using the result of Equation (10), leading to the following approximation:

(11) $$\begin{array}{cc} \mathrm{Softmax}\left(q_{j}k_{\delta j}^{⊤}\right) & \approx \phi \left(q_{j}k_{\delta j}^{⊤}\right)\\ \; & \approx \left[\phi \left(q_{j}^{+}\right)\phi {\left(k_{\delta j}^{+}\right)}^{⊤}+\phi \left(q_{j}^{-}\right)\phi {\left(k_{\delta j}^{-}\right)}^{⊤}\right]\\ \; & -\left[\phi \left(q_{j}^{+}\right)\phi {\left(k_{\delta j}^{-}\right)}^{⊤}+\phi \left(q_{j}^{-}\right)\phi {\left(k_{\delta j}^{+}\right)}^{⊤}\right] \end{array}$$

In Equation (11), the two bracketed terms on the right-hand side correspond to the positive and negative semantic correlation weights, respectively. It can be observed from this equation that this integration not only enables the characterization of negative semantic correlations but also supplements positive semantic correlations, empowering the model to more accurately capture directional and structural features in point cloud data.

The kernel function ϕ(·) in Equation (11) is defined as

(12) $$\phi \left(u_{j}\right)=\left[u_{1}^{t_{1}},\ldots ,u_{M}^{t_{M}}\right]$$

where $t_{1},\cdots ,t_{M}$ are learnable power-exponent scaling coefficients for key feature enhancement. These coefficients form a scaling coefficient row vector $t=[t_{1},t_{2},\ldots ,t_{M}]$, whose calculation is given by

(13) t=1+α·σ(w)

where 1 is an all-one row vector, α controls the upper bound of the learnable exponent, σ(·) denotes the sigmoid function, and $w=[w_{1},w_{2},\ldots ,w_{M}]$ is a learnable parameter vector. Since $\sigma \left(w_{m}\right)\in \left(0,1\right)$, each exponent is bounded by $t_{m}\in \left(1,1+\alpha \right)$. In this work, α is set to 1, so that $t_{m}\in \left(1,2\right)$, which prevents excessively large exponents and improves numerical stability. Equation (13) enables the model to adaptively adjust the power exponent for different feature dimensions, thereby enhancing feature responses in a controlled nonlinear manner. For the power mapping $\phi \left(u\right)=u^{t}$, the input gradient is $\partial \phi \left(u\right)/\partial u=tu^{t-1}$. Since t<2 and the input *u* is non-negative after polarity decomposition, the bounded exponent formulation avoids high-order power operations and serves as an implicit regularization for stable training.

With the derivation of the Equation (11), substituting the row vectors corresponding to $q_{j}^{+}$, $q_{j}^{-}$, $k_{\delta j}^{+}$, $k_{\delta j}^{-}$, $v_{j}^{L}$ and $v_{j}^{R}$ into Equation (2) yields the positive and negative semantic correlation row vectors:

(14) $$o_{j}^{\mathrm{Pos}}=\frac{\phi \left(q_{j}^{+}\right)\sum_{k=1}^{K}\phi {\left(k_{\delta k}^{+}\right)}^{⊤}v_{k}^{L}+\phi \left(q_{j}^{-}\right)\sum_{k=1}^{K}\phi {\left(k_{\delta k}^{-}\right)}^{⊤}v_{k}^{L}}{\phi \left(q_{j}\right)\sum_{k=1}^{K}\phi {\left(k_{\delta k}\right)}^{⊤}+\epsilon }$$

(15) $$o_{j}^{\mathrm{Neg}}=\frac{\phi \left(q_{j}^{+}\right)\sum_{k=1}^{K}\phi {\left(k_{\delta k}^{-}\right)}^{⊤}v_{k}^{R}+\phi \left(q_{j}^{-}\right)\sum_{k=1}^{K}\phi {\left(k_{\delta k}^{+}\right)}^{⊤}v_{k}^{R}}{\phi \left(q_{j}\right)\sum_{k=1}^{K}\phi {\left(k_{\delta k}\right)}^{⊤}+\epsilon }$$

It should be noted that the denominators in Equations (14) and (15) use the complete query and key terms, namely $\phi (q_{j})$ and $\phi (k_{\delta k})$, rather than polarity-specific components. This shared normalization term is designed to balance the positive and negative branches under a unified scale and avoid inconsistent normalization between the two branches. During model training, noise interference is unavoidable, which may cause the denominator in Equation (2) to approach zero, resulting in degraded numerical stability. To mitigate this noise impact, a perturbation term $\epsilon =10^{-6}$ is introduced into the denominators of Equations (14) and (15). This ensures the denominator remains non-zero, thereby enhancing the numerical stability of the computation.

The fusion output component concatenates the positive and negative semantic correlation vectors (outputted by the two branches) via the Concat operation, forming a 1×M-dimensional combination coefficient row vector $s_{j}=\left[o_{j}^{\mathrm{Pos}};o_{j}^{\mathrm{Neg}}\right]$. The combination coefficient row vector $s_{j}$ then undergoes normalization to ensure the sum of its elements equals 1. After all *K* neighboring points in the local neighborhood complete this interaction process, the combination coefficient matrix S is obtained. With the integration of polarity-aware attention, the resulting matrix S not only retains richer spatial geometric information but also strengthens the interaction between key point pairs, which facilitates a more rational allocation of the corresponding weight matrices in the subsequent neighborhood weight set.

### 3.4. Polarity Convolution Kernel Generation

The polarity convolution kernel generation sub-module generates the polarity convolution kernel set κ by leveraging the combination coefficient matrix S and the neighborhood weight set ω. The kernel set κ consists of *K*$C_{in}\times C_{out}$-dimensional polarity convolution kernels ${\left\{\kappa_{ik}\right\}}_{k=1}^{K}$, each designed to model the point cloud features within the local neighborhood. For the *j*-th neighboring point $p_{j}$ in the *K*-neighborhood of the central point $p_{i}$, the corresponding polarity convolution kernel $\kappa_{ij}$ is computed using the combination coefficient row vector $s_{j}$ (associated with $p_{j}$) and the neighborhood weight set $\omega ={\left\{\omega_{m}\right\}}_{m=1}^{M}$:

(16) $$\kappa_{ij}=\sum_{m=1}^{M}s_{jm}\omega_{m}$$

where $s_{jm}$ is the *m*-th element of the combination coefficient row vector $s_{j}$. By iterating over all row vectors in matrix S, we obtain the full polarity convolution kernel set $\kappa ={\left\{\kappa_{ik}\right\}}_{k=1}^{K}$, where each kernel corresponds to a neighboring point in the local neighborhood. The polarity convolution kernel $\kappa_{ij}$ enables adaptive, per-point weighting of the semantic features of neighboring points $p_{j}$. This flexibly encodes the geometric properties of the point cloud neighborhood, adapting to local geometric variations (e.g., planes, edges, and corners), and ensures that the weight assignment aligns with the inherent characteristics of the local structure.

### 3.5. Feature Aggregation

The unstructured nature of point cloud data poses fundamental challenges to the accurate extraction and effective fusion of local geometric features. While convolution kernels can capture diverse local edge information, achieving efficient aggregation of these heterogeneous features to enhance geometric representation capability remains a core priority for advancing point cloud analysis performance. To address this, a novel feature aggregation strategy is adopted in the feature aggregation sub-module. By establishing a one-to-one mapping between the polarity convolution kernel set ${\left\{\kappa_{ik}\right\}}_{k=1}^{K}$ and the semantic features $X_{i}$ of the corresponding neighboring points, a structured fusion method is used to enhance the central point $p_{i}$’s ability to model geometric features. Specifically, the neighborhood set of $p_{i}$ contains *K* neighboring points; for each neighboring point $p_{j}$, its associated feature corresponds to the row vector $x_{ij}$ in the semantic feature matrix $X_{i}$. This vector is paired with a dedicated polarity convolution kernel $\kappa_{ij}$. The kernel $\kappa_{ij}$ deeply integrates geometric prior knowledge with the central point’s features by encoding local geometric relationships, thereby generating more discriminative high-level semantic representations. The feature aggregation process then aggregates the semantic representations of all neighboring points in the neighborhood into the semantic feature of the central point $p_{i}$:

(17) $$y_{i}=\sum_{k=1}^{K}x_{ik}\kappa_{ik}^{T}$$

The semantic feature $y_{i}$ of $p_{i}$ (calculated via Equation (17)) exhibits enhanced representational power, as it synthesizes the geometric features of all neighboring points relative to the central point. By effectively fusing the diverse features of local edge information, $y_{i}$ provides a richer feature representation foundation with robust geometric perception capabilities, laying a strong groundwork for subsequent point cloud semantic segmentation tasks.

## 4. Proposed Structure

**Figure 5:**
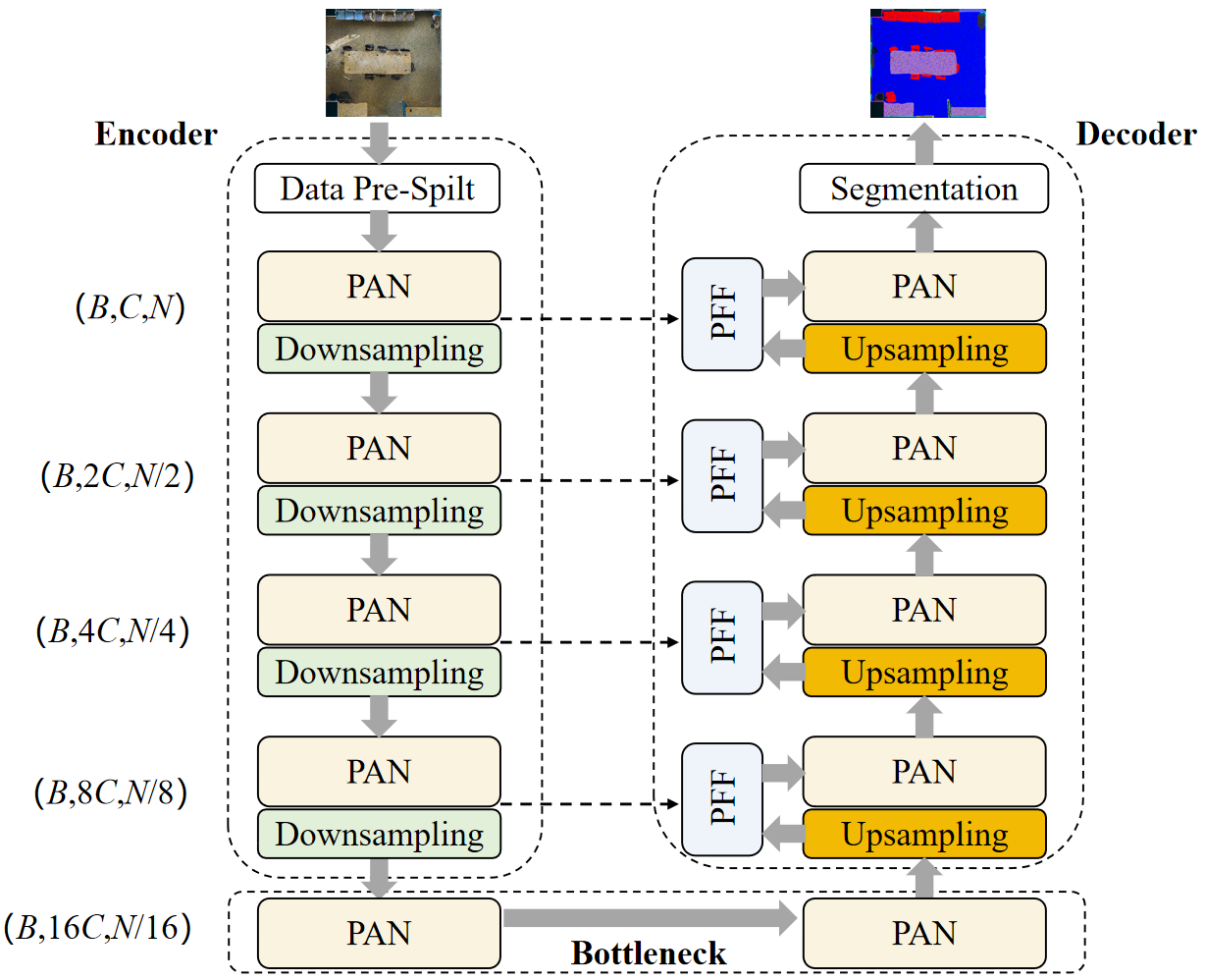
Encoder–decoder architecture with PAN and PFF. In the figure, dashed lines represent feature skip connections, while solid arrows indicate sequential feature transmission.

The overall framework of PA-DFNet is illustrated in Figure 5. This work is extended from the PointNet++ [16] baseline, preserving its hierarchical feature extraction paradigm to efficiently capture the multi-scale spatial information inherent in point clouds. To address the inherent limitations of PointNet++ in three aspects (polarity correlation modeling, feature fusion strategy, and local structure perception), we design targeted enhancement strategies. These modifications compensate for the original framework’s shortcomings in the collaborative learning of point cloud semantic and geometric features.

The specific improvements of PA-DFNet to the PointNet++ architecture are concentrated on the innovative design and replacement of two core modules. On the one hand, the PAN module replaces the MLP in the original framework for feature extraction. During feature transformation, traditional MLPs rely on activation functions (e.g., ReLU) that tend to truncate negative feature components, leading to the loss of the inherent positive and negative semantic correlation information in point clouds. By decoupling the polarity processing pathways of query–key pairs, the PAN module can specifically preserve and model both positive and negative semantic correlations. This effectively mitigates the aforementioned limitation, while strengthening the feature representation capability for both geometric and semantic polarity. On the other hand, the PFF module is introduced to replace the original fixed skip connections. The fixed skip connections employed by PointNet++ lack the ability to dynamically adjust weights based on the semantic importance of features, making it challenging to bridge the semantic gap between the low-level geometric detail features output by the encoder and the high-level semantic abstract features generated by the decoder. By integrating channel attention and spatial attention mechanisms, the PFF module enables adaptive weight calibration and deep aggregation of cross-scale features, not only retaining critical geometric details but also improving semantic consistency.

Additionally, because the PAN module performs feature aggregation within K-nearest neighbor (KNN) local neighborhoods, its complexity is more appropriately analyzed as O(NK) rather than directly compared with global $O(N^{2})$ self-attention. Here, *K* denotes the number of neighboring points selected for each central point. When the neighborhood size *K* is fixed, the computational cost scales linearly with the number of points *N*, while preserving PointNet++’s local–global hierarchical feature learning capability. This markedly enhances the processing efficiency of large-scale point cloud data, eliminating inference latency induced by excessive computational overhead from feature interactions. Following polarity-aware feature extraction via the PAN module and cross-scale dynamic fusion through the PFF module, the network finally generates a comprehensive representation that fuses the spatial positional information and geometric structure features of the point cloud. This comprehensive representation is fed into a segmentation module consisting of fully connected layers for category prediction, which then outputs point-wise semantic segmentation or part segmentation results, delivering precise 3D semantic annotations to support subsequent tasks.

### 4.1. Overall Architecture Design

Both the encoder and decoder of PA-DFNet employ a four-layer stacked structure, as shown in Figure 5. The layer count is chosen with reference to the Point Transformer model [17], striking a balance between feature extraction capability and computational efficiency. The encoder takes raw point cloud data as input, formatted as a matrix (B,C,N), where *B* represents the batch size, *C* is the feature dimension, and *N* is the number of points in the point cloud. Subsequently, the data undergoes initial processing via the Data Pre-Split module, which partitions the data into semantic features X and geometric features P. The initial dimension of X is determined by the input data type. If the input only contains 3D coordinate information, X has an initial dimension of N×3; if the input incorporates additional attributes (e.g., color), the initial dimension of X is adjusted accordingly. P is constructed based on the neighborhood geometry. The neighborhood geometric information adopted herein includes the 3D coordinates of the central point, the relative spatial position of each neighboring point with respect to the central point, and the Euclidean distance between them. Therefore, P is N×7. During downsampling and upsampling, geometric features P transmit the spatial information evolution of the point cloud (from dense to sparse and then back to dense), providing foundational support for the multi-scale feature extraction of the PAN module. Following processing by each PAN module, a sampling operation is performed. During downsampling, the spatial scale of the point cloud is halved iteratively (the number of points is reduced to half its original count, i.e., *N* is reduced to N/2), while the feature dimension is doubled incrementally (*C* is increased to 2C). After successive downsampling steps, the encoder outputs features in the format (B,16C,N/16). Upsampling, the inverse operation of downsampling, restores the point cloud to its original size and recovers the point features via interpolation. At the end of the encoder and the start of the decoder, a Bottleneck layer is incorporated. This layer consists of sequential 1×1 convolution, 3×3 convolution, and 1×1 convolution layers. Each convolution operation is followed by a BN layer and a ReLU activation layer. Its function is to further compress feature dimensions, integrate the multi-level feature information extracted by the encoder, and provide a well-adapted input for the subsequent processing in the decoder. After being processed by the bottleneck layer, the features output by the encoder are fed into the decoder. The decoder first leverages the PFF module to fuse shallow and deep features across different levels. Through a series of convolution operations and attention mechanisms, the PFF module can effectively aggregate cross-level features to bolster feature representation capability. The fused features then sequentially pass through the PAN module and upsampling operations, gradually restoring the point cloud to its original spatial scale, thereby recovering the spatial information of the point cloud. Finally, after being processed by the Segmentation module (composed of fully connected layers), the network outputs the point cloud segmentation prediction.

### 4.2. PFF Model

**Figure 6:**
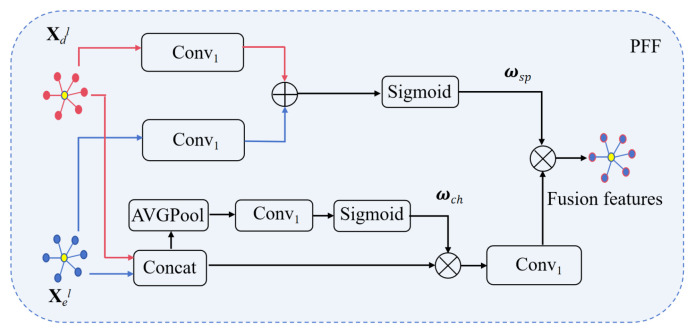
Structural diagram of the PFF integrating spatial and channel attention. The red part represents deep decoder features, while the blue part represents shallow encoder features.

While the fixed skip connections in PointNet++ enable cross-scale feature propagation, a notable semantic gap exists between the low-level geometric features output by the encoder and the high-level semantic abstract features produced by the decoder. Fixed-weight feature concatenation under this setup tends to introduce information redundancy or result in the loss of critical fine-grained details. To address this limitation, we propose the PFF module, as shown in Figure 6. This module leverages attention mechanisms to adaptively calibrate the weights of cross-scale features, thereby facilitating more precise feature aggregation.

The PFF module takes the deep decoder features $X_{e}^{l}$ and shallow encoder features $X_{d}^{l}$ (where *l* denotes the hierarchical level of the features) as inputs. These two feature types are first concatenated along the channel dimension to form the fused feature tensor $\left[X_{e}^{l},X_{d}^{l}\right]$. To enable efficient management of the fused features, the PFF module incorporates a feature calibration mechanism based on global channel information, which sequentially applies average pooling, convolution, and nonlinear mapping operations to $\left[X_{e}^{l},X_{d}^{l}\right]$ to derive global channel weights:

(18) $$w_{ch}=\mathrm{Sigmoid}\left({\mathrm{Conv}}_{1}\left(\mathrm{AVGPool}\left(\left[X_{e}^{l},X_{d}^{l}\right]\right)\right)\right)$$

where AVGPool extracts global statistical information along the channel dimension, ${\mathrm{Conv}}_{1}$ applies a convolution operation to the pooling output to perform feature transformation, and the Sigmoid activation function maps the result to the interval [0,1], assigning a weight to each channel. The global channel weights $w_{ch}$ are then used to calibrate and filter the features:

(19) $$X^{l}={\mathrm{Conv}}_{1}\left(w_{ch}⊙\left[X_{e}^{l},X_{d}^{l}\right]\right)$$

where ⊙ denotes the Hadamard product. Specifically, the fused feature tensor $\left[X_{e}^{l},X_{d}^{l}\right]$ is weighted by $w_{ch}$ according to channel importance and then refined via the ${\mathrm{Conv}}_{1}$ convolutional layer. This process preserves critical features, suppresses redundant channels, and ultimately yields the feature tensor $X^{l}$ that integrates global channel information.

To capture the spatial-dimensional feature correlation between the deep decoder features $X_{e}^{l}$ and shallow encoder features $X_{d}^{l}$, the PFF module further incorporates a global spatial attention mechanism. For the deep decoder features $X_{e}^{l}$ and shallow encoder features $X_{d}^{l}$, the module first extracts their respective local spatial features (e.g., object edges and part interfaces) via the ${\mathrm{Conv}}_{1}$ convolutional layer. Next, it fuses the spatial information of these two feature types using matrix addition and then generates the global spatial weight through the Sigmoid activation function:

(20) $$w_{sp}=\mathrm{Sigmoid}\left({\mathrm{Conv}}_{1}\left(X_{e}^{l}\right)+{\mathrm{Conv}}_{1}\left(X_{d}^{l}\right)\right)$$

Using the global spatial weight $w_{sp}$, global spatial information is integrated into the feature $X^{l}$ (which already embeds global channel information) via the Hadamard product:

(21) $${\hat{X}}^{l}=w_{sp}⊙X^{l}$$

At this stage, the feature ${\hat{X}}^{l}$ output by the PFF module achieves dynamic balancing and integration between the low-level geometric detail features of the encoder and the high-level semantic features of the decoder. This integration enhances the segmentation accuracy of critical regions (e.g., edges) in point cloud segmentation. Compared with the fixed skip connections in PointNet++, the computational complexity of the PFF module undergoes a modest increase, approximately $O\left(NC_{l}^{2}\right)$, where $C_{l}$ denotes the feature dimension of the *l*-th layer. However, since the complexity scales linearly with the number of points *N* in the point cloud, the module remains applicable to large-scale point cloud segmentation tasks.

### 4.3. Loss Function Design

In point cloud classification and semantic segmentation tasks, it is essential to simultaneously evaluate two critical performance metrics: the category-level alignment of the entire point cloud (for classification) and the per-point semantic label consistency (for segmentation). Cross-entropy serves as a well-established metric for quantifying the discrepancy between two probability distributions. By formulating the ground-truth per-point labels and the predicted per-point classifications of the point cloud as two distinct probability distributions, a per-point cross-entropy loss can be employed to unify the optimization objectives of both classification and segmentation tasks. Notably, the classification task can be framed as a special case of segmentation, where the label of a single representative point encodes the category of the entire point cloud.

For a given point $p_{i}$ within the point cloud, its ground-truth label $g_{z}\left(i\right)$ is represented using a one-hot scheme, specifically, $g_{z}\left(i\right)=1$ if $p_{i}$ belongs to the *z*-th point cloud category and $g_{z}\left(i\right)=0$ otherwise. Let ${\hat{g}}_{z}\left(i\right)$ denote the probability that the neural network assigns $p_{i}$ to the *z*-th category. In this context, the ground-truth label $g_{z}\left(i\right)$ and the predicted probability ${\hat{g}}_{z}\left(i\right)$ can be interpreted as two probability characterizations of $p_{i}$. Consequently, cross-entropy is adopted to characterize the prediction loss of the network for $p_{i}$, which is defined as

(22) $$L_{\mathrm{CE}}=-\sum_{i=1}^{Z}g_{z}\left(i\right)\log{\hat{g}}_{z}\left(i\right)$$

where *Z* represents the total number of categories for point cloud classification or semantic segmentation. Since Equation (22) quantifies the prediction loss for an individual point $p_{i}$, $L_{\mathrm{CE}}$ is also referred to as the point-wise cross-entropy loss. By penalizing the deviation between predicted labels and ground-truth annotations on a per-point basis, $L_{\mathrm{CE}}$ not only enforces the category prediction consistency of the entire point cloud in classification tasks but also enables fine-grained calibration of each point’s semantic label in the segmentation tasks. Although a large neighborhood weight set ω can accommodate more weight matrices, these matrices (initialized with random values) tend to converge to similar states during training, leading to weight matrix diversity degradation. The core function of weight matrices is to aggregate features from neighboring points. Distinct weight matrices generate differentiated feature aggregation rules (e.g., some prioritize capturing edge structures, while others focus on planar regions). If multiple weight matrices become highly similar, their corresponding kernel functions will tend to be homogeneous, ultimately resulting in the learning of only redundant or analogous feature patterns, which fails to capture the multi-scale and multi-morphological characteristics of point clouds. To mitigate this issue, a weight matrix regularization loss $L_{\mathrm{corr}\;}$ is designed. First, we compute the mean of all weight matrices in the neighborhood weight set:

(23) $$\bar{\omega }=\frac{1}{M}\sum_{m=1}^{M}\omega_{m}$$

where *M* denotes the total number of weight matrices in the set ω. Next, the correlation coefficient between any two distinct weight matrices can be calculated as

(24) $$r\left(\omega_{i},\omega_{j}\right)=\frac{{\langle \omega_{i}-\bar{\omega },\omega_{j}-\bar{\omega }\rangle }_{F}}{\sqrt{{\langle \omega_{i}-\bar{\omega },\omega_{i}-\bar{\omega }\rangle }_{F}}\sqrt{{\langle \omega_{j}-\bar{\omega },\omega_{j}-\bar{\omega }\rangle }_{F}}}$$

where ${\langle A,B\rangle }_{F}$ represents the Frobenius inner product of conformable matrices A and B, defined as the sum of the element-wise products of all corresponding entries in A and B. Using the correlation coefficient, the weight matrix regularization loss can be constructed as

(25) $$L_{\mathrm{corr}}=\sum_{\omega_{i},\omega_{j}\in \omega ,i\neq j}\left|r(\omega_{i},\omega_{j})\right|$$

This loss sums the absolute values of the correlation coefficients for all unique pairs of weight matrices (with i>j to avoid redundant calculations), thereby penalizing the scenario where any two weight matrices become highly similar. Minimizing $L_{\mathrm{corr}\;}$ forces different weight matrices to maintain distinct characteristics, enabling the model to learn richer and more diverse feature patterns. Meanwhile, by quantifying the deviation of each weight matrix from the global mean $\bar{\omega }$, this loss effectively enhances the distributional diversity of the weight set, mitigates linear dependency issues, and ensures the diversity of generated kernel functions. Finally, by combining the cross-entropy loss $L_{\mathrm{CE}}$ and the weight matrix regularization loss $L_{\mathrm{corr}\;}$, the total loss function is derived as

(26) $$L_{\mathrm{total}}=L_{\mathrm{CE}}+\lambda L_{\mathrm{corr}}$$

where λ is a hyperparameter that balances the relative contributions of the two loss components. Since no closed-form analytical solution exists for directly computing the optimal value of λ, we determine the value that maximizes model performance via an empirical grid search over the validation set.

## 5. Experiments and Discussion

### 5.1. Experimental Setup

#### 5.1.1. Datasets

To comprehensively evaluate the performance of PA-DFNet on point cloud classification and semantic segmentation tasks, we selected three widely adopted and highly recognized benchmark datasets in the point cloud domain: ModelNet40, ShapeNet, and S3DIS. ModelNet40 is used to evaluate object-level point cloud classification performance, ShapeNet is used to evaluate part-level segmentation capability, and S3DIS is used to evaluate scene-level indoor semantic segmentation performance. These three datasets cover different levels of point cloud understanding, thereby enabling a more comprehensive validation of the effectiveness and generalization ability of PA-DFNet. Subsequently, we perform task-specific model training and evaluation on these datasets, tailored to the unique characteristics of each task.

**Figure 7:**
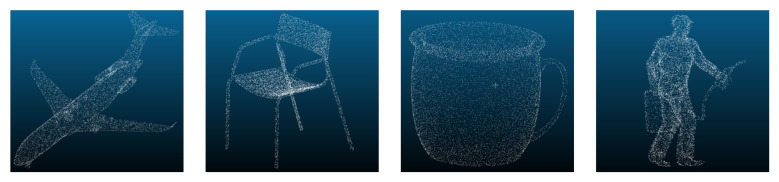
ModelNet40 point cloud samples.

ModelNet40 is a classic benchmark dataset for 3D object classification and recognition, released by Princeton University in 2015 [38]. It serves as a core benchmark for evaluating algorithmic performance in this domain. The dataset encompasses 40 categories of common objects (e.g., airplanes, cars, chairs, and tables), with a total of 12,311 computer-aided design (CAD) models, including 9843 samples for training and 2468 samples for testing. Here, CAD refers to computer-aided design, which represents digitally constructed 3D object models. In our experiments, each CAD model is represented as a point cloud for task input. To ensure experimental fairness when comparing against baselines such as PointNet++ and DGCNN, we uniformly sample 1024 points as input for all models. This configuration aligns with the standard input specifications in the related literature, eliminating performance comparison biases introduced by variations in input scale. Figure 7 presents example objects from the ModelNet40 dataset. These instances visually demonstrate the diverse object categories covered by ModelNet40 and their corresponding point cloud representations, providing an intuitive reference for subsequent point cloud classification and other tasks conducted on this dataset.

ShapeNet is a widely used benchmark dataset for 3D shape analysis and point cloud part segmentation [39]. Different from ModelNet40, which focuses on object-level classification, ShapeNet provides fine-grained part annotations for objects from multiple categories, enabling the evaluation of part-level geometric understanding. In this study, ShapeNet is adopted to further assess the segmentation capability and generalization performance of PA-DFNet on object parts with diverse geometric structures.

S3DIS (Stanford 3D Indoor Spaces) is a leading benchmark dataset for indoor scene semantic segmentation, collected and annotated by Stanford University [40]. Covering 6 independent indoor regions (Areas 1–6), the dataset comprises a total of 271 room scenes. A domain-standard data split protocol is adopted: Area 5 is exclusively used as the test set to ensure objective evaluation, while Areas 1–4 and 6 are merged to form the training set, establishing a standardized verification system with separated training and test partitions. In terms of data content, S3DIS encompasses 13 high-frequency indoor semantic categories, including architectural structures (e.g., walls, floors, and ceilings) and furniture items (e.g., tables, chairs, sofas, and bookshelves), with a total of approximately 273 million points. For semantic annotation, S3DIS provides 3D spatial coordinates, RGB color information, and corresponding semantic labels for each point. To ensure annotation reliability, all semantic labels undergo manual point-wise verification and cross-validation, resulting in a final annotation accuracy of over 95%. This effectively mitigates the interference of manual annotation errors on experimental results and provides a high-reliability ground truth reference for the accuracy assessment of indoor scene point cloud semantic segmentation tasks.

#### 5.1.2. Evaluation Metrics

For point cloud classification and segmentation tasks, task-specific evaluation metrics are employed to quantify model performance, enabling fair and consistent comparisons across different algorithms.

For point cloud classification tasks, the core evaluation metrics are overall accuracy (OA) and mean accuracy (mAcc). OA quantifies the overall prediction correctness across all categories, computed as

(27) $$\mathrm{OA}=\frac{1}{Z}\sum_{z=1}^{Z}{\mathrm{TP}}_{z}$$

where ${\mathrm{TP}}_{z}$ denotes the number of true positives for the z-th category (i.e., samples belonging to class z that are correctly predicted as class z). The mAcc is calculated by first computing the prediction accuracy of each individual category and then averages these per-category accuracies. This metric reflects the performance balance across all categories. It is defined as

(28) $$\mathrm{mAcc}=\frac{1}{Z}\sum_{z=1}^{Z}\frac{{\mathrm{TP}}_{z}}{{\mathrm{TP}}_{z}+{\mathrm{FN}}_{z}}$$

where ${\mathrm{FN}}_{z}$ represents the number of false negatives for the z-th category (i.e., samples belonging to class z that are incorrectly predicted as non-class z). For point cloud part and semantic segmentation tasks, the mean Intersection over Union (mIoU) serves as the core evaluation metric, which is built upon the Intersection over Union (IoU) for each individual category. The IoU for the z-th category is defined as

(29) $${\mathrm{IoU}}_{z}=\frac{{\mathrm{TP}}_{z}}{{\mathrm{TP}}_{z}+{\mathrm{FP}}_{z}+{\mathrm{FN}}_{z}}$$

where ${\mathrm{FP}}_{z}$ denotes the number of false positives for the *z*-th category (i.e., samples not belonging to the *z*-th category that are incorrectly predicted as positive samples of this category). As evident from Equation (29), ${\mathrm{IoU}}_{z}$ is the ratio of the intersection to the union of the predicted and ground-truth instances for the *z*-th category, directly reflecting the segmentation accuracy of that category. The mIoU is calculated by first computing the IoU for each individual category and then averaging these values across all categories. This metric characterizes the overall average segmentation performance across all global categories, and its formula is

(30) $$\mathrm{mIoU}=\frac{1}{Z}\sum_{z=1}^{Z}{\mathrm{IoU}}_{z}$$

In terms of metric interpretation, larger values of mAcc and OA indicate higher overall or category-averaged prediction accuracy in classification tasks. Conversely, a larger mIoU value corresponds to better category-level or global segmentation precision in segmentation tasks.

#### 5.1.3. Training Configuration

Our training configuration is detailed in Table 1. The experimental environment is built on the Ubuntu 22.04 LTS operating system, which provides a stable and efficient foundation for deep learning tasks. The CPU employed is an AMD Ryzen 5 Pro 4650G, featuring multi-threaded processing capabilities to assist in executing various computational tasks throughout the training process. For GPU acceleration, we use two NVIDIA GeForce RTX 4070 16G graphics cards. Their robust parallel computing capabilities and combined 32GB video memory effectively expedite the training workflow of deep learning models.

The system is equipped with 32GB of RAM. We adopt Python 3.7 as the programming language, paired with the PyTorch 2.1.0 deep learning framework—its extensive toolkits and user-friendly development workflow facilitate rapid model construction and debugging. Additionally, we leverage CUDA 12.4 for training acceleration, which maximizes the computational performance of the GPUs.

The configurations of point cloud classification and segmentation tasks across different datasets are summarized in Table 2. For classification experiments on the ModelNet40 dataset, we set the learning rate to 0.1 and the batch size to 32. The SGD optimizer is configured with an initial learning rate of 0.1 and a momentum coefficient of 0.9. The model is trained for 350 epochs to ensure precise identification of 3D model categories. For semantic segmentation experiments on the S3DIS dataset, the learning rate is set to 0.05 and the batch size to 16. We use the SGD optimizer (with a momentum coefficient of 0.9 and a weight decay coefficient of 0.0001) and train for 120 epochs, enabling the model to segment different target objects within indoor scenes. In all point cloud experiments, a unified data augmentation pipeline is adopted to enhance model generalization: random rotation of the point cloud around the z-axis, scaling of the point cloud at a variable ratio, and addition of jitter noise to point coordinates. This strategy equips the trained model with stronger robustness and generalization capabilities.

### 5.2. Key Parameter Settings

As noted previously, PA-DFNet requires the pre-configuration of two critical hyperparameters: the number of neighborhood points *K* and the number of weight matrices *M*. The selection of these parameters directly impacts both the computational efficiency and the overall performance of PA-DFNet. Therefore, we take the ModelNet40 dataset as the benchmark for the classification task. Following the experimental environment and configuration specified in Section 5.1.3, we conduct classification experiments on ModelNet40 to analyze how the performance of PA-DFNet varies with different values of *K* (neighborhood point count) and *M* (weight matrix count).

#### 5.2.1. Selection of the Number of Neighborhood Points

In the feature learning process for point cloud data, the choice of the neighborhood point count *K* directly dictates the quality of local feature extraction and the computational efficiency of the model. On the one hand, the neighborhood serves as the region for modeling local geometric structures and semantic correlations within the point cloud. The number of points (i.e., *K*) directly determines the richness of contextual information that a single central point can aggregate, which in turn influences the ability of features to represent fine-grained local details. On the other hand, a larger *K* (i.e., more neighborhood points) requires each central point to perform feature interaction operations with a greater number of neighborhood points, which directly degrades the model’s inference speed and training efficiency.

When fixing the number of weight matrices *M*, we conduct classification experiments on the ModelNet40 dataset using different values of *K*, and the corresponding OA and mAcc metrics are presented in Table 3. Table 3 illustrates that as *K* increases, both evaluation metrics exhibit a trend of initial improvement followed by a subsequent decline. When *K* is too small, insufficient neighborhood structural information hinders the full extraction of local geometric and semantic features from the point cloud. Conversely, when *K* is too large, the model tends to overfit to redundant information about the overall shape, while neglecting fine-grained structural features at the boundaries of adjacent neighborhoods. Additionally, a larger *K* increases computational complexity and amplifies interference from redundant information.

Point cloud classification and segmentation tasks demand both fine-grained segmentation within individual categories and high overall recognition accuracy across the entire scene. When K=20, OA reaches its optimal value of 93.1%, while mAcc achieves 90.5%, which is only 0.1% lower than the peak mAcc observed at K=16, thus remaining at a high level. At this setting, the overall segmentation integrity of all categories in the scene is further enhanced. Therefore, considering both global model performance and the segmentation requirements of practical scenarios, we select 20 as the optimal parameter value for *K*.

#### 5.2.2. Selection of the Number of Weight Matrices

The choice of the number of weight matrices *M* in the PAN module is crucial for balancing the segmentation accuracy and computational complexity of PA-DFNet. Therefore, it is necessary to investigate how *M* impacts the overall performance of the network. In our experiments, we fix K=20 (the optimal neighborhood point count determined in Section 5.2.1) and conduct classification tasks on the ModelNet40 dataset using different values of *M*. The corresponding mAcc and OA results are summarized in Table 4.

From Table 4, we observe that model accuracy exhibits a trend of initial improvement followed by a subsequent decline as *M* (the number of weight matrices) increases. Weight matrices serve as the foundational components that enable the model to adapt to diverse geometric structures (e.g., planes, edges, and corners) within local point cloud neighborhoods. When M=2, the limited diversity of weight matrices fails to cover the rich geometric–semantic patterns in point clouds, restricting the model’s feature representation capability. This results in a modest performance (OA = 91.4%, mAcc = 89.3%). When M=4, the enhanced diversity of weight matrices strengthens the model’s feature expression capability (OA = 92.0%, mAcc = 90.1%). When M=8, the weight matrices achieve optimal alignment with the local geometric–semantic correlations in the point cloud. This configuration fully captures the structural characteristics of diverse regions while avoiding excessive redundant parameters, thus reaching peak performance (OA = 93.1%, mAcc = 90.5%). When M=16, an excessive number of weight matrices introduces redundant parameters, increasing model complexity. This makes the model prone to overfitting to noise or trivial local details in the training data (rather than learning generalized geometric features), leading to reduced test accuracy (OA = 92.6%, mAcc = 89.8%). Additionally, a larger *M* imposes a heavier computational burden on the model. In conclusion, to balance classification accuracy and computational efficiency, we select M=8 as the optimal number of weight matrices. This choice ensures the model can represent diverse local geometric–semantics while mitigating the risk of overfitting.

### 5.3. Point Cloud Classification Experiment Analysis

To validate the effectiveness of PA-DFNet in point cloud classification tasks, we conducted training and testing experiments on the ModelNet40 dataset, using OA and mAcc as the core evaluation metrics. To ensure strict experimental fairness, we followed the standard input configurations of mainstream baseline models (e.g., PointNet++ and DGCNN). Specifically, the input point cloud size for PA-DFNet and all comparison models (including DGCNN, PointConv, and KPConv) was uniformly set to 1024 points, with the input format retained as the original point cloud (Point). All experimental parameters were configured according to Section 5.1, and PA-DFNet employed the optimal hyperparameters determined earlier: neighborhood point count K=20 and weight matrix count M=8. The classification results of all methods on the ModelNet40 dataset are summarized in Table 5. The performance values of the comparison methods are cited directly from their original published papers, and bolded values indicate the best performance for the corresponding metrics.

These comparative experiments fully validate the effectiveness of PA-DFNet for point cloud classification tasks. Through its polarity-aware and dynamic feature fusion mechanisms, PA-DFNet extracts point cloud features more efficiently, enabling dual optimization of both global classification accuracy and category-level performance balance. This work thus provides a more competitive solution for point cloud classification tasks.

### 5.4. Point Cloud Part Segmentation Experiment Analysis

To further evaluate the effectiveness of the proposed method on fine-grained point cloud understanding tasks, we conduct point cloud part segmentation experiments on the ShapeNetPart dataset. In this experiment, PA-DFNet is constructed based on the PointNet++ framework. Specifically, the proposed PAN module is introduced to replace the original MLP-based local feature extraction operation in PointNet++, and the PFF module is employed to enhance cross-level feature fusion between the encoder and decoder. The resulting network is compared with several representative point cloud part segmentation methods. The quantitative results are reported in Table 6.

As shown in Table 6, PA-DFNet achieves an overall mIoU of 86.5% and a class-average mIoU of 83.9% on the ShapeNetPart dataset. Compared with PointNet, PointNet++, and DGCNN, the proposed method improves the overall mIoU by 2.8, 1.4, and 1.3 percentage points, respectively. In terms of class-average mIoU, PA-DFNet also improves the performance by 3.5, 2.0, and 1.6 percentage points over PointNet, PointNet++, and DGCNN, respectively. These results demonstrate that replacing the MLP-based local feature extraction in PointNet++ with polarity-aware feature modeling can effectively enhance fine-grained part-level representation.

From the category-wise results, PA-DFNet obtains the best or highly competitive IoU values in several categories, such as bag, laptop, pistol, and skateboard. In particular, the proposed method achieves IoU scores of 85.7%, 95.9%, 84.7%, and 80.5% on these categories, respectively. These categories usually contain complex local structures or ambiguous part boundaries, indicating that the proposed PAN module is beneficial for capturing discriminative local geometric relationships, while the PFF module helps preserve detailed part-level information during encoder–decoder feature propagation.

**Figure 8:**
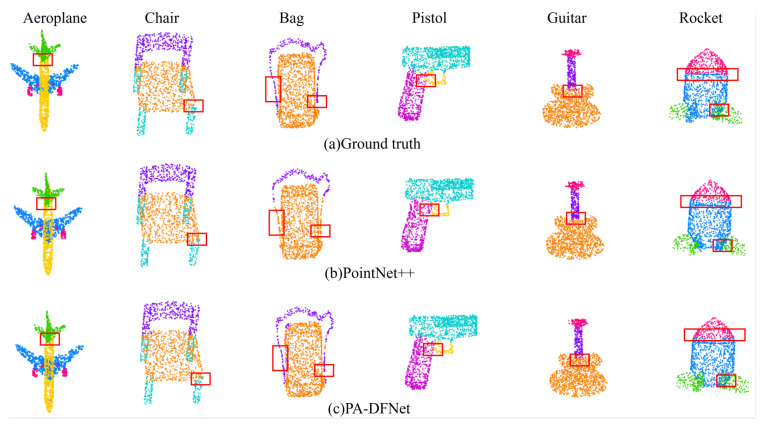
Visualization comparison of point cloud part segmentation results on the ShapeNetPart dataset. The red boxes highlight representative local regions where segmentation differences are observed.

To provide a more intuitive comparison, several representative visualization results are shown in Figure 8. PointNet++ is used as the baseline method for visual comparison because PA-DFNet is developed from the PointNet++ framework. The red boxes highlight local regions with noticeable segmentation differences. Compared with the PointNet++ baseline, PA-DFNet produces more complete and consistent part segmentation results, especially around fine-grained structures and part boundaries.

### 5.5. Point Cloud Semantic Segmentation Experiment Analysis

Point cloud semantic segmentation involves predicting a semantic label for each point by leveraging the spatial structure and shape information of the point cloud. Given the unstructured nature and inherent semantic complexity of point clouds, this task presents substantial technical challenges. To validate the effectiveness of PA-DFNet for point cloud semantic segmentation, we conducted training and testing experiments on the S3DIS dataset, following the experimental environment and configuration specified in Section 5.1.3. Consistent with the optimal parameter settings determined in Section 5.2, we set the neighborhood point count K=20 and the number of weight matrices M=8.

Table 7 presents the semantic segmentation performance comparison between PA-DFNet and several classical methods on the S3DIS dataset. All performance metrics of the comparative methods in this table are cited from their original literature. Note that only PointNet and PointCNN provide mAcc values. From these performance metrics, PointNet, as a foundational model for direct point cloud processing, avoids information loss during preprocessing. However, its reliance on global-only feature aggregation leaves it incapable of capturing local neighborhood details, resulting in a modest mIoU of 41.1% and mAcc of 48.9%. PointNet++ builds upon PointNet by introducing hierarchical sampling and local feature aggregation, which strengthens its ability to extract multi-scale geometric features and boosts mIoU to 50.6%. Nevertheless, its fixed feature propagation and aggregation mechanism lack the capacity to adaptively fuse cross-scale features, and it fails to fully account for polarity correlations, leading to suboptimal segmentation at object edges. DGCNN leverages dynamic graph convolution to capture local geometric relationships, enabling adaptation to local point cloud structures. However, the high computational complexity of graph convolution and ambiguous neighborhood selection make it prone to feature confusion in complex multi-category indoor scenes, yielding a low mIoU of 47.0% and weak inter-object discrimination capability.

PointCNN enhances geometric feature extraction via the local perception properties of convolution operators, delivering stable segmentation performance for large planar objects (mAcc = 64.9%). However, the limited adaptability of convolution operations to non-Euclidean structures results in insufficient segmentation accuracy for irregular fine-grained components (e.g., chair legs and picture edges), leading to an overall mIoU of only 57.3%. RandLA-Net focuses on efficient large-scale point cloud processing. It balances efficiency and accuracy through random downsampling and lightweight feature extraction, achieving an mIoU of 62.4%. Yet, it sacrifices the refinement of fine-grained features for efficiency, which degrades segmentation performance at the junctions of small objects or components. KPConv adopts deformable convolution to dynamically adjust kernel shapes, improving its ability to fit local geometric structures and achieving strong segmentation performance in complex geometric regions (e.g., curved surfaces and edges) with an mIoU of 65.4%. That said, dynamic convolution incurs high computational overhead, and the model lacks targeted designs for polarity correlations and cross-scale feature fusion, leaving room for further improvement in overall accuracy.

Our proposed PA-DFNet achieves an mIoU of 66.2% and an mAcc of 72.8%, outperforming all comparative models. This superior performance stems from two key module designs. The PAN module effectively restores semantic polarity correlations (e.g., the association between “table” and “tabletop objects”), addressing the edge-blurring issue that arises when traditional models overlook negative semantic information. The PFF module enables adaptive fusion of multi-scale features. It not only preserves low-level geometric details (e.g., the edge structures of tables and chairs) but also enhances the high-level semantic consistency across different object categories within the room. Together, these mechanisms drive comprehensive improvements in both mIoU and mAcc.

**Figure 9:**
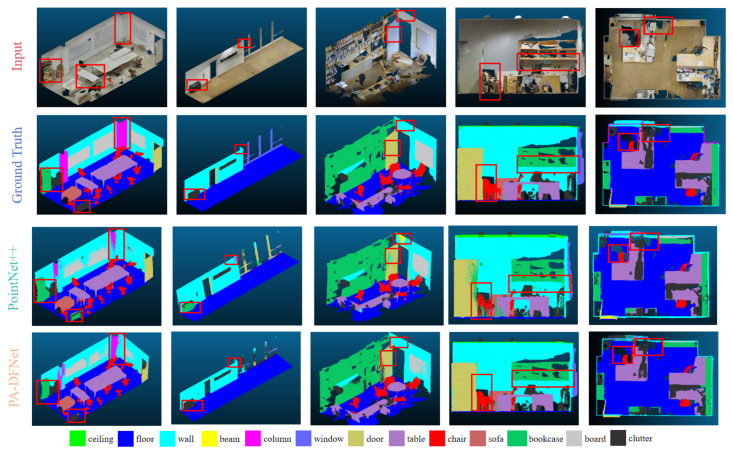
Qualitative comparison of semantic segmentation results on the S3DIS Area-5 dataset. Each column corresponds to a representative indoor scene, and the four rows show the input point cloud, ground truth, PointNet++ prediction, and PA-DFNet prediction, respectively. Red boxes highlight local regions where segmentation differences are observed. The color legend indicates the semantic categories used for visualization.

To intuitively demonstrate the semantic segmentation performance of PA-DFNet, we randomly selected five typical indoor room point clouds from Area 5 of the S3DIS dataset for visual analysis of segmentation results, as shown in Figure 9. In Figure 9, each column corresponds to one of five representative indoor scenes: a meeting room, a corridor, a bookshelf-equipped workspace, a bookshelf-equipped office, and a multi-person office workspace. These scenes cover common spatial layouts and object categories in indoor environments, enabling a comprehensive assessment of the model’s segmentation performance across diverse scenarios. Figure 9 consists of four rows of visualization results. The first row displays the original point cloud with RGB color information. While PA-DFNet does not utilize color data for semantic segmentation, the colored point cloud clearly illustrates the indoor scene layout, serving as an objective reference for subsequent qualitative and quantitative evaluation of segmentation performance. The second row shows the ground-truth segmentation, where the point cloud is colored via a standard “label-color mapping” scheme. For example, all points belonging to the chair category (semantic label = 8) are uniformly rendered red. This one-to-one color-category correspondence clearly delineates the spatial contours and semantic boundaries of each object, enabling rapid visualization of the true object distribution in the scene. The third and fourth rows present the segmentation outputs of PointNet++ and PA-DFNet, respectively, with points colored according to their predicted semantic labels.

From the semantic segmentation outputs of PointNet++ (third row) and PA-DFNet (fourth row) in Figure 9, both methods are capable of performing semantic segmentation on diverse indoor scenes. When compared to the ground-truth label visualization (second row), the predicted labels of both models exhibit a high degree of alignment with the true semantic distributions. Specifically, both methods can not only accurately segment large continuous structures (e.g., cyan-blue walls and blue floors) but also effectively segment small objects in the scene (e.g., green bookshelves, purple desks, and red tables/chairs). By examining the regions highlighted with red boxes across all indoor scenes in Figure 9, PA-DFNet demonstrates superior semantic segmentation accuracy in these localized areas, further confirming its advantage over PointNet++ in fine-grained segmentation tasks.

To further quantify the semantic segmentation performance of PA-DFNet, Table 8 presents the per-category IoU results of relevant methods across Area 5 of the S3DIS dataset. The segmentation accuracy metrics of the comparison methods in Table 8 are reported according to the corresponding original papers or commonly used benchmark reports. In Area 5 of S3DIS, the Beam category contains an extremely small number of samples, making it difficult for models to learn reliable semantic features for this class. Therefore, following common reporting practice, the Beam category is omitted in Table 8.

Comparing the IoU results across the 12 reported categories, PA-DFNet achieves strong performance on large planar structure categories, with 93.9% for Ceiling, 98.5% for Floor, and 82.1% for Wall. These results indicate that PA-DFNet can effectively segment regular geometric structures. For furniture-related categories, PA-DFNet achieves the best IoU on table, with 87.9%, and obtains competitive results on Chair and Sofa, with 87.8% and 73.7%, respectively. Compared with PointNet++, whose IoU values are 69.2% for table, 71.1% for Chair, and 46.2% for Sofa, PA-DFNet shows clear improvements in modeling complex semantic objects. For fine-grained or edge-related categories, PA-DFNet obtains 23.5% on Column and 58.8% on Window, which are comparable to RandLA-Net with 24.7% on Column and 62.3% on Window. These results demonstrate that PA-DFNet preserves effective segmentation capability for small-scale and structurally detailed categories while maintaining strong overall semantic representation.

### 5.6. Ablation Experiments

The ablation experiments in this section were conducted on the ModelNet40 dataset to verify the effectiveness of each module in PA-DFNet. All experimental parameters (including learning rate, batch size, training epochs, and optimizer type) strictly adhered to the classification task configuration specified in Table 2. The experimental environment was fixed to the hardware and software specifications outlined in Table 1, and we used the optimal parameter settings determined earlier: neighborhood point count K=20 and weight matrix number M=8.

#### 5.6.1. Necessity of Point Cloud Polarity Recovery

To verify the necessity of point cloud polarity recovery, we selected the minimalist point cloud processing framework PointNet as the baseline model for comparative experiments. As a pioneering work in point cloud feature extraction, PointNet adopts a core paradigm of “Multilayer Perceptron (MLP) + global pooling” for feature extraction. Its minimalist architecture minimizes confounding factors from multi-modular structures (e.g., hierarchical learning and skip connections, as implemented in PointNet++), allowing us to isolate and evaluate the independent impact of the PAN module’s point cloud polarity recovery mechanism on point cloud classification performance.

**Figure 10:**
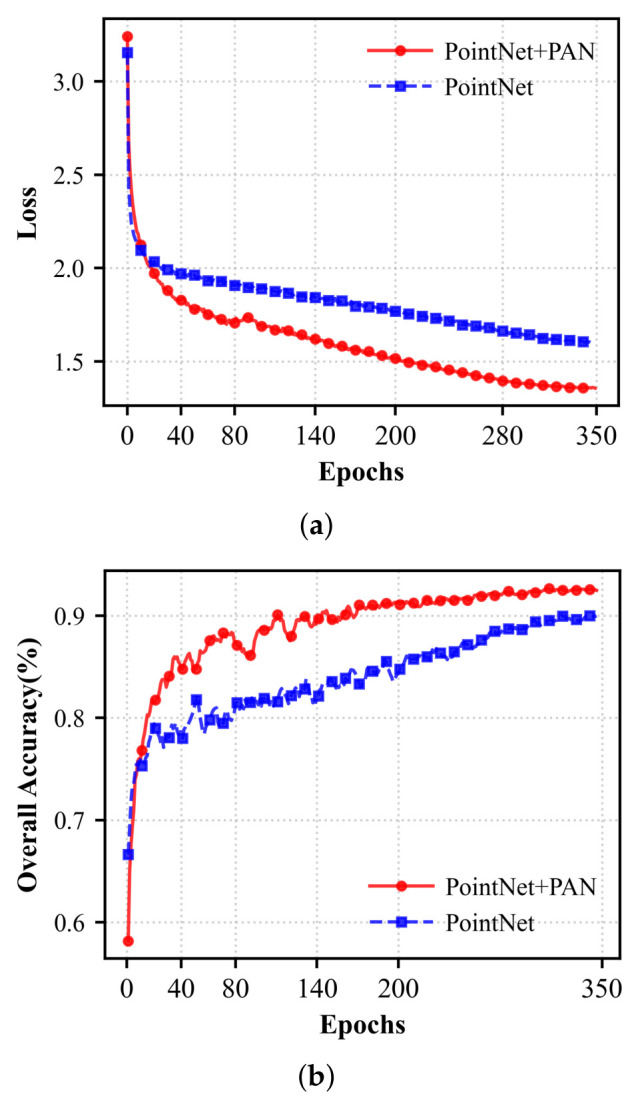
Training loss and test OA performance of PointNet and PointNet+PAN on ModelNet40. (**a**) Analysis of training loss. (**b**) Analysis of test OA.

Using the ModelNet40 dataset and adhering to the experimental parameters specified in Section 5.1, we recorded the training loss and test set OA changes for the PointNet model (blue curve) and the PointNet+PAN model (red curve, which integrates the PAN module). The results are shown in Figure 10. From the training loss curve in Figure 10a, it can be observed that in the early training stages, due to the additional overhead for polarity decomposition and attention calculation introduced by the PAN module, the loss of PointNet+PAN is slightly higher than that of PointNet. As training epochs increase, the loss of PointNet+PAN drops rapidly, while the loss reduction rate of PointNet slows down gradually. By the late training stage, PointNet+PAN achieves significantly lower loss and converges more stably. This indicates that the polarity-aware attention mechanism improves the efficiency of model parameter optimization and reduces loss fluctuations caused by redundant features.

From the test-set OA curve in Figure 10b, both models show continuous improvement in test OA as training progresses. PointNet+PAN’s OA surges rapidly from 54% in the early stages of training, eventually stabilizing at 92.7%, which is a 3.5 percentage point improvement over PointNet (89.2%). The core driver of this accuracy gain lies in the fact that PointNet+PAN, via its polarity recovery mechanism, can more precisely distinguish positive and negative polarity correlations in point clouds, enabling efficient feature extraction and optimizing the training process.

In summary, the experimental results in Figure 10 validate the necessity of the polarity-aware attention mechanism in point cloud feature extraction. Polarity recovery not only strengthens the model’s ability to capture fine-grained polarity correlations but also makes feature learning more targeted through efficient linear attention calculations, ultimately enabling the model to outperform the baseline PointNet.

#### 5.6.2. Ablation Experiments on Module Combinations

To validate the independent effectiveness and synergistic complementarity of the PAN and PFF modules in PA-DFNet, we conducted ablation experiments on the PointNet++ framework using the ModelNet40 dataset as the benchmark. Four differentiated network configurations were designed to systematically analyze how module combinations impact model performance: (1) original PointNet++ architecture (serving as the baseline model); (2) replacing the MLP in the core structure of PointNet++ with the PAN module; (3) replacing the fixed skip connections in the core structure of PointNet++ with the PFF module; and (4) replacing both the MLP with the PAN module and the fixed skip connections with the PFF module in the core structure of PointNet++. The performance comparison results of models under different module combinations are shown in Table 9.

Experiment 1 (Baseline model): It adopts the original PointNet++ architecture, which relies solely on traditional hierarchical feature learning. It achieves an OA of 90.7% and an mAcc of 88.3%.

Experiment 2 (PAN module only): Relative to the baseline (Experiment 1), it improves OA by 2.2% and mAcc by 1.8%. This indicates that the PAN module enhances the expressive capability of local geometric features precisely by modeling the positive and negative polarity correlations within the neighborhoods of data points.

Experiment 3 (PFF module only): When compared with Experiment 1, it yields a 0.5 percentage point improvement in OA and a 1.1 percentage point improvement in mAcc. This validates that in object interaction scenarios, the PFF module is superior to the original fixed skip connections in terms of feature fusion efficiency. It efficiently fuses features from the shallow encoder and deep decoder, strengthens the complementarity of multi-level features, and thus alleviates the feature balance challenge inherent in hierarchical networks.

Experiment 4 (joint integration of PAN and PFF): It achieves the optimal performance, with an OA of 93.1% and an mAcc of 90.5%, representing 2.4 percentage points and 2.2 percentage points of improvement in OA and mAcc, respectively, compared to the baseline. In this configuration, the PAN module first completes refined extraction of semantic and geometric polarity features, and then, the PFF module dynamically fuses multi-level features. The results of Experiment 4 demonstrate that PA-DFNet further enhances network performance through the synergy of local feature detail enhancement (enabled by PAN) and cross-scale feature alignment (enabled by PFF). This validates the complementarity of the two core modules in the PA-DFNet architecture, fully confirming the rationality of its design.

#### 5.6.3. Ablation Experiments on PAN Sub-Component Configurations

As shown in Table 10, the baseline variant without polarity decomposition obtains 63.4% mIoU and 70.1% mAcc. Using either the positive or negative polarity branch improves the segmentation performance, indicating that polarity-aware correlation modeling is beneficial for local feature extraction. When the positive and negative branches are jointly used, the mIoU increases to 65.2%, demonstrating the complementary effect of the two polarity branches. Furthermore, introducing positional encoding improves the mIoU from 65.2% to 65.7%, suggesting that explicit geometric cues help enhance local structural representation. The full PAN configuration with the learnable power function achieves the best performance, with 66.2% mIoU and 72.8% mAcc. These results verify the effectiveness of polarity decomposition, positional encoding, and adaptive nonlinear scaling in PAN.

#### 5.6.4. Ablation Experiments on PFF Sub-Component Configurations

Table 11 reports the ablation results of different PFF configurations. Compared with the fixed skip-connection baseline, using channel attention alone improves the mIoU from 64.7% to 65.4%, while using spatial attention alone further improves the mIoU to 65.6%. This indicates that channel attention and spatial attention contribute to feature recalibration from different perspectives. When both attention branches are combined, the full PFF module achieves the best performance, with 66.2% mIoU and 72.8% mAcc. These results demonstrate that the performance gain of PFF comes from the joint modeling of channel-wise feature importance and spatial structural responses, rather than from simple module stacking.

### 5.7. Robustness Tests

Existing point cloud analysis methods generally underperform in scenarios with sparse point clouds (low point counts) or noise interference. To verify the robustness and generalization capability of the proposed PA-DFNet, two targeted tests were conducted on the ModelNet40 dataset: a point density robustness test and a noise robustness test. In these experiments, all key parameters related to classification tasks strictly followed the configuration specified in Table 2 (for classification tasks). The hardware and software environment adhered to the specifications outlined in Table 1, and PA-DFNet used the parameter settings determined earlier: neighborhood point count K=20 and weight matrix number M=8.

#### 5.7.1. Point Density Robustness Experiment

In real-world scenarios, point cloud density is highly non-uniform. For example, LiDAR systems in outdoor environments typically generate locally dense point clouds (on the surfaces of nearby objects) alongside globally sparse point clouds (on the edges of distant scenes), which is caused by object occlusion (e.g., buildings or trees) or signal attenuation at long ranges. Additionally, variations in sampling accuracy across different sensor models further amplify density fluctuations, which directly undermine a model’s ability to capture geometric features.

**Figure 11:**
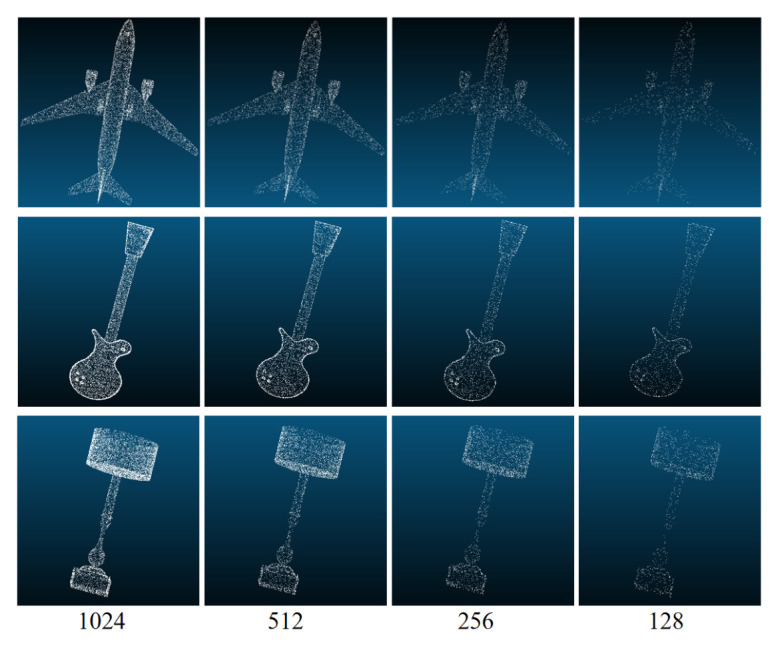
Visualization of point clouds with varying point densities for object representation.

To simulate such real-world conditions and validate PA-DFNet’s robustness to density variations, we employed a random downsampling strategy: we discarded original point cloud data at different ratios to construct experimental samples with varying levels of sparsity. Figure 11 provides an intuitive visualization of the point clouds (for three representative object categories: airplane, guitar, and desk lamp) under different point-count configurations.

**Figure 12:**
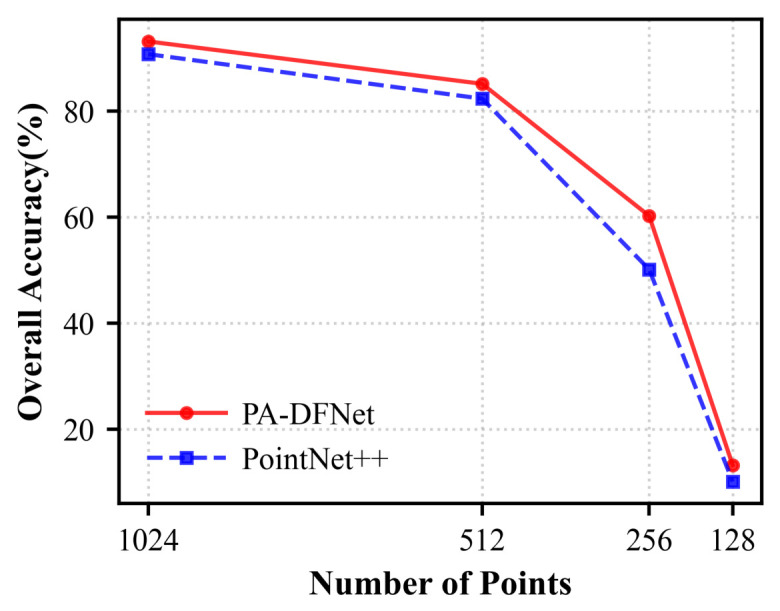
Classification accuracy of PA-DFNet and PointNet++ under different point densities.

To further quantify the robustness to point density variations, we evaluated the classification accuracy of PA-DFNet and PointNet++ across point counts ranging from 128 to 1024, as illustrated in Figure 12. It can be seen from Figure 12 that the OA of both models decreases as point cloud sparsity increases, but PA-DFNet consistently outperforms PointNet++ across all configurations. At 1024 points, PA-DFNet reaches 93.1% OA, compared to 90.7% for PointNet++ (a 2.4 percentage point lead). At 512 points, the OA of PA-DFNet remains 85.1%, while PointNet++ drops to 82.3% (expanding the lead to 2.8 percentage points). At 256 points, PA-DFNet maintains 60.2% OA, whereas PointNet++ only reaches 50.1% (the lead widens to 10.1 percentage points). Even at extreme sparsity (128 points), PA-DFNet still retains a performance advantage over PointNet++. Point cloud recognition under low-density conditions imposes higher demands on a network’s learning capability. PA-DFNet meets this challenge by restoring negative semantic correlations and fusing shallow (fine-grained geometric) and deep (high-level semantic) features, which generates more discriminative feature representations, thus demonstrating strong robustness.

#### 5.7.2. Noise Robustness Experiment

To simulate real-world noise interference, we introduced Gaussian noise (with zero mean) into the 3D coordinates (X, Y, Z) of the original point cloud (excluding auxiliary information such as color). The noise variances were set to 0.02, 0.04, 0.06, 0.08, and 0.1 (with the unit of $m^{2}$). As the variance increases, the point cloud undergoes greater perturbation, and the noise interference on the model intensifies. The experiment used the accuracy drop rate *P* as the quantitative metric for noise robustness, which is calculated as

(31) $$P=\frac{OA_{\mathrm{noise}}-OA}{OA}\times 100\%$$

where OA is the overall accuracy of the model (on the test set) without noise, and ${\mathrm{OA}}_{\mathrm{noise}}$ denotes the overall accuracy after adding noise.

The experimental results are presented in Table 12. As observed, the OA of all models decreases with increasing noise variance, which is consistent with the adverse impact of noise on model performance. Compared to PointNet++ and DGCNN, PA-DFNet demonstrates superior anti-noise capability. At a variance of 0.02, PA-DFNet’s drop rate (−1.2%) is lower than that of PointNet++ (−1.7%) and DGCNN (−1.5%). At a variance of 0.04, PA-DFNet’s drop rate (−1.8%) still outperforms PointNet++ (−2.8%) and DGCNN (−2.1%). For higher variances (0.06, 0.08, and 0.1), PA-DFNet exhibits the smallest performance degradation (drop rates: −2.9%, −6.1%, and −7.2%, respectively), significantly outperforming PointNet++ and DGCNN.

PA-DFNet’s noise robustness stems from three key design elements: its embedding layer constructs rich low-level geometric relationships, its encoding layer enables effective learning of high-level features, and its adaptive fusion module amplifies the weights of salient information in noisy scenarios. This allows PA-DFNet to effectively mitigate performance degradation as noise levels increase, validating its superiority in processing noisy point clouds.

### 5.8. Model Complexity Test

Model complexity is a core metric for evaluating network performance. In this section, we conduct a comprehensive complexity analysis of PA-DFNet alongside mainstream point cloud models (e.g., PointNet, PointNet++, DGCNN, and Point Transformer) across four dimensions: parameter count (in millions, M), model size (in megabytes, MB), inference speed (in frames per second, FPS), and classification accuracy (OA).

All experiments were uniformly deployed on an Ubuntu 22.04 system, with two NVIDIA GeForce RTX 4070 16G GPUs serving as hardware acceleration units. Model inference acceleration was implemented using the PyTorch 2.1.0 framework and CUDA 12.4. The test was conducted on per-frame point cloud inputs of 1024 points. Inference speed (FPS) was measured via continuous inference experiments on fixed-scale point clouds. The inference task was run 1000 consecutive times; the first 100 runs were discarded to eliminate unstable data from hardware warm-up, and the final FPS was calculated using the average runtime of the remaining 900 runs. The experimental results are summarized in Table 13.

From Table 13, PA-DFNet achieves an OA of 93.1%, outperforming PointNet (89.2%), PointNet++ (90.7%), and DGCNN (92.9%). Its accuracy is nearly indistinguishable from Point Transformer (93.2%), demonstrating strong high-precision feature recognition capability. In terms of parameter count and model size, PA-DFNet uses 8.7 M parameters (a 53% reduction relative to Point Transformer’s 18.5 M) and has a 32.9 MB model size (35.5% smaller than Point Transformer’s 51 MB). This efficiency stems from its polarity-aware linear attention mechanism. Unlike Point Transformer’s self-attention, which incurs high computational overhead from “point-to-all” interactions, PA-DFNet balances robust feature perception with parameter efficiency. For inference speed, PA-DFNet achieves 102.4 FPS, which is far faster than Point Transformer (47.9 FPS) and comparable to PointNet++ (98.7 FPS). Specifically, PA-DFNet achieves approximately 2.14 times the inference speed of Point Transformer while maintaining only a 0.1% OA gap, indicating a more favorable balance between accuracy and efficiency. Compared with PointNet++, PA-DFNet improves OA by 2.4% while maintaining a slightly higher FPS, further demonstrating its practical computational efficiency. This means it maintains high accuracy while delivering efficient inference, indicating potential for efficient inference on large-scale point clouds.

In summary, PA-DFNet excels in the synergistic optimization of parameter efficiency, inference speed, and classification accuracy achieved through its polarity-aware linear attention mechanism. Future research can focus on the design of lightweight modules to further reduce computational costs while preserving the model’s accuracy advantages.

## 6. Conclusions

To address the issues of missing polarity correlations, inefficient feature fusion, loss of fine-grained details, and excessive computational complexity of self-attention mechanisms in existing point cloud models, this paper proposes the PA-DFNet network based on PointNet++, which realizes the refined extraction of polarity features and adaptive fusion of cross-scale features via two core modules, namely PAN and PFF. Validated on mainstream benchmark datasets, including ModelNet40, ShapeNet, and S3DIS, the model achieves excellent performance in both point cloud classification and semantic segmentation tasks. Specifically, on the ModelNet40 classification task, PA-DFNet improves OA and mAcc by 2.4% and 2.2%, respectively, compared with PointNet++. The model also demonstrates strong robustness to sparse point clouds and noise as well as high computational efficiency. Theoretically, this study establishes a novel modular design paradigm for multi-task learning on point clouds. Practically, the model provides a promising basis for future point cloud applications such as autonomous driving and 3D reconstruction, and its modular architecture enables flexible migration across different tasks. This study still has limitations including limited dataset coverage, empirical setting of partial parameters and insufficient adaptability to class-imbalanced scenarios. Future research will expand the validation scenarios, explore adaptive parameter learning, integrate with Transformer architectures, and optimize loss functions, so as to further enhance the model’s generalization ability and applicability in diverse scenarios.

## Figures and Tables

**Table 1:** Experimental environment configuration.

Experimental Environment	Setting
Operating system	Ubuntu 22.04 LTS
CPU	AMD Ryzen 5 Pro 4650G
GPU	NVIDIA GeForce RTX 4070 16G × 2
Operational memory	32 GB
Graphics memory	32 GB
Programming language	Python 3.7
Deep learning framework	PyTorch 2.1.0
Training acceleration	CUDA 12.4

**Table 2:** Experimental parameters of network training.

Parameters	ModelNet40	S3DIS
Learning Rate	0.1	0.05
Batch Size	32	16
Optimizer	SGD	SGD
Epochs	350	120
Experiment	Classification	Segmentation

**Table 3:** Effect of different *K* values on model performance.

*K*	OA/%	mAcc/%
5	92.4	89.5
10	92.6	90.1
15	92.9	**90.6**
20	**93.1**	90.5
25	92.5	89.1

Note: Bold values indicate the best performance for each metric.

**Table 4:** Selection of the number of weight matrices *M*.

*M*	OA/%	mAcc/%
2	91.4	89.3
4	92.0	90.1
8	**93.1**	**90.5**
16	92.6	89.8

Note: Bold values indicate the best performance for each metric.

**Table 5:** Classification performance comparison on ModelNet40.

Model	Input	Points	Normal	OA/%	mAcc/%
VoxNet	Voxel	–	No	85.9	83.0
MVCNN	Image	–	No	–	90.1
PointNet	Point	1k	No	89.2	86.2
PointNet++	Point	1k	No	90.7	88.3
DGCNN	Point	1k	No	92.9	90.2
LDGCNN	Point	1k	No	92.3	90.3
PointConv	Point	1k	No	92.5	–
PointCNN	Point	1k	No	92.2	88.1
KPConv	Point	1k	No	92.4	–
PointASNL	Point	1k	No	92.9	–
PAConv	Point	1k	No	**93.9**	–
PCT ^†^	Point	1k	Yes	93.7	**90.6**
PA-DFNet	Point	1k	No	93.1	90.5

Note: PCT ^†^ denotes Point Cloud Transformer; Bold values indicate the best performance for each metric.

**Table 6:** Category-wise IoU comparison on ShapeNetPart dataset.

Model	IoU for different categories								
	aero.	bag	cap	car	chair	earph.	guitar	knife	lamp
PointNet	83.4	78.7	82.5	74.9	89.6	73.0	91.5	85.9	80.8
PointNet++	82.4	79.0	87.7	77.3	90.8	71.8	91.0	85.9	83.7
DGCNN	84.0	83.4	86.7	77.8	90.6	74.7	91.2	87.5	82.8
LDGCNN	84.0	83.0	84.9	78.4	90.6	74.4	91.0	88.1	83.4
PointASNL	84.1	84.7	87.9	79.7	**92.2**	73.7	91.0	87.2	84.2
PAConv	84.3	85.0	**90.4**	79.7	90.6	**80.8**	**92.0**	**88.7**	82.2
PCT ^†^	**85.0**	82.4	89.0	**81.2**	91.9	71.5	91.3	88.1	**86.3**
Ours	83.5	**85.7**	88.8	78.1	90.3	76.6	91.7	88.0	81.0
Model	IoU for different categories								
	laptop	motor	mug	pistol	rocket	skate	table	Cls.mIoU	mIoU
PointNet	95.3	65.2	93.0	81.2	57.9	72.8	80.6	80.4	83.7
PointNet++	95.3	71.6	94.1	81.3	58.7	76.4	82.6	81.9	85.1
DGCNN	95.7	66.3	94.9	81.1	63.5	74.5	82.6	82.3	85.2
LDGCNN	95.8	67.4	94.9	82.3	59.2	76.0	81.9	82.2	85.1
PointASNL	95.8	**74.4**	95.2	81.0	63.0	76.3	83.2	83.3	86.1
PAConv	**95.9**	73.9	94.7	**84.7**	**65.9**	**81.4**	**84.0**	**84.6**	86.1
PCT ^†^	95.8	64.6	**95.8**	83.6	62.2	77.6	83.7	–	86.4
Ours	**95.9**	71.1	94.1	**84.7**	61.9	80.5	80.5	83.9	**86.5**

Note: PCT ^†^ denotes Point Cloud Transformer; Bold values indicate the best performance for each metric.

**Table 7:** Point cloud semantic segmentation results on the S3DIS Area-5 dataset.

Model	Protocol	mIoU/%	mAcc/%
PointNet	Area-5	41.1	49.0
DGCNN	Area-5	47.0	–
PointNet++	Area-5	57.3	–
PointCNN	Area-5	57.3	63.9
SPGraph	Area-5	58.0	66.5
PointConv	Area-5	60.0	–
PointWeb	Area-5	60.3	66.6
HPEIN	Area-5	61.9	68.3
RandLA-Net	Area-5	62.4	–
PointASNL	Area-5	62.6	68.5
FPT	Area-5	63.5	70.2
PointMLP	Area-5	64.2	71.5
MinkowskiNet	Area-5	65.4	71.7
KPConv	Area-5	65.7	71.8
PAConv	Area-5	65.9	72.1
PA-DFNet	Area-5	**66.2**	**72.8**

Note: Bold values indicate the best performance for each metric.

**Table 8:** Quantitative IoU comparison on S3DIS (Area 5) (%).

Model	Ceil.	Floor	Wall	Col.	Win.	Door	Table	Chair	Sofa	Book.	Board	Clut.
PointNet	87.4	97.8	71.2	9.2	52.1	16.3	58.2	48.6	3.2	48.3	39.0	36.2
PointNet++	89.4	97.7	75.4	1.8	58.3	19.5	69.2	71.1	46.2	57.4	58.7	41.6
PointCNN	85.9	97.8	79.4	17.6	28.8	62.1	70.4	80.6	39.7	66.7	62.1	56.7
RandLA-Net	91.1	95.6	80.2	24.7	**62.3**	47.7	76.2	83.7	60.2	71.1	65.7	53.8
MinkowskiNet	91.8	97.7	**86.2**	**34.1**	48.9	62.4	81.6	89.8	47.2	74.9	70.4	58.6
KPConv	92.8	97.3	82.4	23.9	58.0	**69.0**	81.5	**91.0**	**75.4**	**75.3**	66.7	**58.9**
PA-DFNet	**93.9**	**98.5**	82.1	23.5	58.8	65.5	**87.9**	87.8	73.7	69.3	**71.6**	57.5

Note: Ceil., Col., Win., Book., and Clut. denote Ceiling, Column, Window, Bookcase, and Clutter, respectively. Bold values indicate the best performance for each metric.

**Table 9:** Ablation study on module combinations in PA-DFNet.

Number	PointNet++	PAN	PFF	OA (%)	mAcc (%)
1	✓	–	–	90.7	88.3
2	✓	✓	–	92.9	90.1
3	✓	–	✓	91.2	89.4
4	✓	✓	✓	**93.1**	**90.5**

Note: Bold values indicate the best performance for each metric.

**Table 10:** Ablation experiments on PAN sub-component configurations on the S3DIS Area-5 dataset.

Method	No Polarity	Positive	Negative	PE	Power	mIoU/%	mAcc/%
A1	✓	–	–	–	–	63.4	70.1
A2	–	✓	–	–	–	64.1	70.7
A3	–	–	✓	–	–	64.5	71.0
A4	–	✓	✓	–	–	65.2	71.6
A5	–	✓	✓	✓	–	65.7	72.2
A6	–	✓	✓	✓	✓	**66.2**	**72.8**

“No Polarity” denotes the baseline variant without positive-negative polarity decomposition. “Positive” and “Negative” denote the positive and negative polarity branches, respectively. “PE” denotes positional encoding, and “Power” denotes the learnable power function. A6 corresponds to the full PAN configuration used in PA-DFNet. Bold values indicate the best performance for each metric.

**Table 11:** Ablation experiments on PFF sub-component configurations on the S3DIS Area-5 dataset.

Method	Fixed Skip Connection	Channel Attention	Spatial Attention	mIoU/%	mAcc/%
B1	✓	–	–	64.7	70.5
B2	–	✓	–	65.4	71.3
B3	–	–	✓	65.6	71.5
B4	–	✓	✓	**66.2**	**72.8**

Note: Bold values indicate the best performance for each metric.

**Table 12:** Noise robustness comparison.

Noise Variance	PointNet++ (%)	DGCNN (%)	PA-DFNet (%)
0.02	−1.7	−1.5	**− 1.2**
0.04	−2.8	−2.1	**−1.8**
0.06	−6.8	−3.2	**−2.9**
0.08	−9.0	−6.6	**−6.1**
0.10	−11.8	−12.0	**−7.2**

Note: Bold values indicate the best performance for each metric.

**Table 13:** Network complexity comparison.

Model	Parameters (M)	Model Size (MB)	OA (%)	FPS
PointNet	3.4	13.6	89.2	**125.5**
PointNet++	5.6	22.4	90.7	98.7
DGCNN	3.5	13.6	92.9	82.1
Point Transformer	18.5	51	**93.2**	47.9
PA-DFNet	8.7	32.9	93.1	102.4

Note: Bold values indicate the best performance for each metric.

## Data Availability

The data presented in this study are openly available in GitHub at https://github.com/kaishaoxia2-1/PA-DFNet-For-PointCloud-Segmentation (accessed on 1 February 2026).

## Abbreviations

The following abbreviations are used in this manuscript:

PA-DFNet	Polarity-Aware Attention and Feature Dynamic Fusion Network
PAN	Polarity-Aware Network
PFF	Point Cloud Feature Dynamic Fusion
CNN	Convolutional Neural Network
MLP	Multilayer Perceptron
OA	Overall Accuracy
mAcc	Mean Accuracy
IoU	Intersection over Union
mIoU	Mean Intersection over Union
